# Diagnostic oral microbiota signatures for gastric cancer and associations with carcinogenic signaling pathways

**DOI:** 10.1080/20002297.2026.2613531

**Published:** 2026-01-11

**Authors:** Yeon-Hee Kim, Il Ju Choi, Keun Won Ryu, Young-Il Kim, Zeba Praveen, Mi Kyung Kim

**Affiliations:** a Cancer Epidemiology Branch, Division of Cancer Epidemiology and Prevention, National Cancer Center, Goyang, Gyeonggi-do, Republic of Korea; b Center for Gastric Cancer, National Cancer Center Hospital, National Cancer Center, Goyang, Gyeonggi-do, Republic of Korea

**Keywords:** Carcinogenesis, diagnosis, gastric cancer, *Lautropia*, machine learning, *Megasphaera*, microbial markers, oral microflora, saliva, *Ralstonia*

## Abstract

**Background/Objective:**

Gastric cancer (GC) is a major cause of cancer mortality worldwide. We evaluated whether oral microbiota could be sensitive, specific, and non-invasive markers for early GC detection.

**Materials and methods:**

Saliva samples were analyzed using *16S* rRNA sequencing, and oral microbial markers were validated using an internal validation dataset. Machine learning was used to identify key genera, and functional associations were inferred using Kyoto Encyclopedia of Genes and Genomes pathway and ortholog analyses. Blood samples were also collected, and plasma cytokines were quantified by enzyme-linked immunosorbent assay (ELISA) for pathway-level interpretations.

**Results:**

Eight genera—*Lautropia*, *Megasphaera*, *Ralstonia*, *Pseudomonas*, *Peptostreptococcus*, *Anaerovorax*, *Fusobacterium*, and *Neisseria*—were validated as diagnostic microbial markers (area under the receiver operating characteristic curve [AUC] = 0.91). *Megasphaera* and *Ralstonia* were enriched in GC, whereas *Lautropia* was depleted and associated with reduced risk. These genera may be functionally linked to pathways involved in GC progression, including NF-κB, IL-6, STAT3, TGF-β1, and Smad2/3. The proposed classification method effectively identified early-stage and tumor-marker–negative GCs, underscoring its clinical translation potential.

**Conclusions:**

Oral microbial markers, including *Ralstonia*, *Megasphaera*, and *Lautropia*, may serve as non-invasive diagnostic markers for GC and may be related to carcinogenic signaling activity.

## ARTICLE HIGHLIGHTS

- Oral microbiota profiles in saliva can serve as highly accurate, non-invasive diagnostic markers for gastric cancer.
- Specific taxa, including *Ralstonia*, *Megasphaera*, and *Lautropia*, appear to be associated with gastric cancer and related to carcinogenic signaling pathways.
- The diagnostic model effectively detects early-stage and tumor-marker–negative gastric cancer, supporting its clinical utility.

## Introduction

Gastric cancer (GC) ranks fifth in incidence and fourth in mortality, underscoring its global health burden [1]. Early diagnosis markedly improves prognosis, with 5-year survival rates reaching 92.6%, whereas late-stage GC is associated with a 20% survival rate [2,3]. In Korea, biennial endoscopic screening for individuals over 40 years of age has been implemented since 2005, contributing to earlier detection and a gradual decline in GC-related mortality [4,5]. However, endoscopy requires specialised equipment and trained personnel, limiting its scalability for population-wide screening [6], highlighting the need for simpler, cost-effective, and non-invasive diagnostic alternatives.

Gastric microbiota has attracted research attention since *Helicobacter pylori* was identified as a major GC risk factor [7]. Although *H. pylori* infects more than half the global population, only 1–3% of infected individuals develop GC [3,8–10]. Notably, the risk persists even after *H. pylori* eradication, suggesting that additional broader microbial communities or host-related factors may be at play [10,11]. Recently, increasing efforts have been directed to unravel the role of oral microbiota in GC [12]. As the second most diverse microbial community after the gut microbiota, and given the suggested translocation of oral microbes to the gastrointestinal tract, oral microbiota may be involved with GC pathogenesis [13]. Indeed, oral-origin bacteria, such as *Fusobacterium*, *Peptostreptococcus*, and *Veillonella*, have been detected in gastric mucosa of patients with GC, suggesting a potential oral–gastric microbial pathogenic link [14–16]. While saliva collection offers a convenient and non-invasive alternative to endoscopic or faecal sampling [17], few studies have examined the diagnostic relevance of oral microbiota in GC [6,13,18–23]. These studies identified approximately 40 taxa potentially linked to GC, including *Streptococcus*, *Neisseria*, *Prevotella*, and *Porphyromonas*; however, most lacked validation and mechanistic understanding, which hampered their translational potential. Machine learning-based approaches have revealed oral microbial markers linked to multiple cancers, including GC [22]. Furthermore, mounting evidence suggests that gastric microbial dysbiosis can activate oncogenic signalling pathways via cytokine-mediated mechanisms, such as through signal transducer and activator of transcription 3 (STAT3) phosphorylation [24].

The present study aimed to identify and validate oral microbial genera associated with GC using *16S* rRNA sequencing and machine learning. Additionally, we explored the functional relevance of these markers in gastric carcinogenesis by integrating pathway-based functional inference and plasma cytokine validation.

## Materials and methods

### Study participants

Patients with GC and healthy controls (HCs) were recruited from the same hospital under identical clinical protocols to minimise potential environmental or procedural bias, and main clinical characteristics and variables, including age, sex, height, weight, smoking status, alcohol consumption (drinker), body mass index (BMI), cancer stage, and *N* stage, were collected (Table 1). BMI was calculated as weight (kg) divided by the square of height (m^2^). Participants were classified into three groups based on smoking and alcohol consumption: never, former, and current users. Cancer stages were categorised from Stage 1 to Stage 4; whereas *N* stages were classified from N0 to ≥N1. Serum tumour marker levels, including carcinoembryonic antigen (CEA), carbohydrate antigen 19-9 (CA19-9), and carbohydrate antigen 72-4 (CA72-4), were obtained from routine clinical laboratory tests and retrospectively extracted from electronic medical records.

**Table 1.** Demographics, and clinical characteristics of the participants included in this study.

Variable	Discovery data set			Validation data set		
	Gastric cancer (GC) (*n* = 77)	Healthy control (HC) (*n* = 477)	*p-value* ^a^	GC (*n* = 36)	HC (*n* = 190)	*p-value* ^a^
**Age**	59.97 ± 9.19	57.89 ± 9.41	0.0317	62.03 ± 6.84	57.21 ± 8.42	0.0008
<30	0(0.00%)	11(2.28%)		0(0.00%)	4(2.11%)	
30~39	1(1.30%)	14(2.90%)		0(0.00%)	4(2.11%)	
40~49	10(13.0%)	35(7.25%)		2(5.56%)	5(2.63%)	
50~59	17(22.1%)	184(38.1%)	0.0070	10(27.8%)	105(55.3%)	0.0255
60~69	38(49.4%)	208(43.1%)		20(55.6%)	61(32.1%)	
70~79	11(14.3%)	30(6.21%)		4(11.1%)	10(5.26%)	
>= 80	0(0.00%)	1(0.21%)		0(0.00%)	1(0.53%)	
**Sex**						
Female	53(68.8%)	303(62.7%)	3.50 × 10^−7^	32(88.9%)	112(59.0%)	3.71 × 10^−7^
Male	24(31.2%)	180(37.3%)		4(11.1%)	78(41.1%)	
**BMI** ^b^	24.80 ± 3.76	23.99 ± 3.21	0.1168	25.41 ± 3.11	23.84 ± 3.07	0.0036
<18.5	1(1.30%)	13(2.69%)		0(0.00%)	4(2.11%)	
18.5~22.9	23(29.9%)	182(37.7%)	0.4033	6(16.7%)	75(39.5%)	0.0397
23~24.9	24(31.2%)	118(24.4%)		14(38.9%)	56(29.5%)	
>25	29(37.7%)	170(35.2%)		16(44.4%)	55(28.9%)	
**Smoker** ^c^						
Non smoker	41(53.3%)	319(66.1%)		14(38.9%)	119(62.6%)	
Former smoker	20(26.0%)	122(25.3%)	0.0087	13(36.1%)	55(29.0%)	2.29 × 10^−7^
Current smoker	14(18.2%)	34(7.04%)		8(22.2%)	15(7.89%)	
Unknown	2(2.60%)	8(1.66%)		1(2.78%)	1(0.53%)	
**Alcohol consumption (Drinker** ^d^ ^**)**^						
Non drinker	44(57.1%)	277(57.4%)		22(61.1%)	106(55.8%)	
Former drinker	22(28.6%)	121(25.1%)	0.8416	10(27.8%)	59(31.1%)	0.9487
Current drinker	9(11.7%)	66(13.7%)		3(8.33%)	19(10.0%)	
Unknown	2(2.60%)	19(3.93%)		1(2.78%)	6(3.16%)	
**Stage** ^e^						
1A & 1B	58(75.3%)			31(86.1%)		
2A & 2B	4(5.19%)			3(8.33%)		
3A & 3B	8(10.4%)			1(2.78%)		0.2737^f^
4	3(3.90%)			0(0.00%)		
Unknown	4(5.19%)			1(2.78%)		
***N* stage** ^g^						
N0	29(38.2%)			16(44.4%)		
≥N1	19 (25.0%)			2(5.56%)		0.0619^f^
Unknown	28(36.8%)			18(50.0%)		
***H.pylori*** ^h^						
Positive (+)	49(63.6%)			25(69.4%)		1.0000^f^
Negative (−)	22(28.6%)			11(30.6%)		
**Tumour marker**						
CA19-9 (U/mL)^i^	17.7 ± 68.0			10.0 ± 5.56		0.8611^f^
CA72-4 (U/mL)^j^	4.71 ± 3.92			4.01 ± 2.32		0.4686^f^
CEA (ng/mL)	2.76 ± 2.24			3.94 ± 2.56		0.0130^f^
**Marker status**						
All negative (−)^k^	50(64.9%)			19(52.8%)		0.3040^f^
≥1 positive (+)^l^	27(35.1%)			17(47.2%)		

a p-value was computed using Kruskal-Wallis test for continuous variables and chi-square test for categorical variables.

b Body matrix index.

c A smoker is defined as someone who has smoked over 400 cigarettes, with former smokers having quit and non-smokers having not.

d A current drinker consumes alcohol, a former drinker has quit, and a non-drinker has never consumed alcohol.

e In gastric cancer staging, A stages mean the cancer is confined to the stomach layers, while B stages indicate it has spread to nearby lymph nodes. Stages 1 to 4 represent the cancer's progression.

f The p-values were calculated using a chi-square test to compare GC in the discovery dataset with GC in the validation dataset. Tumour marker negativity was defined as CA19-9 < 37 U/mL, CA72-4 < 6.9 U/mL, and CEA < 2.5 ng/mL for non-smokers (<5.0 ng/mL for smokers). Patients were categorised as either.

g It represents the step of cancer spread to nearby lymph nodes.

h *H.pylori* test results of 107 out of 113 GC.

i CA19-9 results of 112 out of 113.

j CA72-4 results of 102 out of 113.

k All markers negative or 1.

l ≥1 marker positive based on CEA, CA19-9, and CA72-4 levels.

### Saliva and blood collection

Baseline saliva (unstimulated whole saliva) samples were collected after at least 1 h of fasting, during which the participants refrained from eating, drinking, or tooth brushing. Before collection, the participants gently accumulated saliva for 1 min and provided approximately 5 mL into a sterile tube. The samples were then aliquoted into 1.5-mL tubes and stored at −80 °C for future analysis. Blood samples were drawn from the antecubital vein using Vacutainer K2 EDTA tubes (BD, Franklin Lakes, NJ, USA) after a 12-h fast. The samples were centrifuged at 1,000 *× g* for 20 min and 4 °C to separate the plasma, buffy coat, and red blood cells, all of which were subsequently stored at −80 °C.

### DNA isolation from saliva samples and *16S* rRNA sequencing

Genomic DNA was extracted from 500 μL of saliva using the Fast DNA Spin Kit (MP Biomedicals, Solon, OH, USA) according to the manufacturer’s protocol. DNA concentration and purity were assessed using a Qubit dsDNA BR Kit (Life Technologies, Carlsbad, CA, USA). The V4 region of *16S* was amplified using fusion primers 515F and 806 R, as recommended by Illumina (San Diego, CA, USA). Polymerase chain reaction (PCR) was performed with an initial denaturation at 95 °C for 3 min, followed by 25 cycles at 95 °C for 30 s, 55 °C for 30 s, and 72 °C for 30 s, with a final extension at 72 °C for 5 min. Amplicons were confirmed via agarose gel electrophoresis, purified using CleanPCR (CleanNA, Waddinxween, The Netherlands), and pooled in equal concentrations. Product quality was verified with a Bioanalyzer 2100 (Agilent Technologies, Santa Clara, CA, USA). Sequencing and library preparation were performed by CJ Bioscience (Seoul, Republic of Korea), an accredited facility that follows rigorous contamination control procedures, including reagent blanks and quality cheques at each processing stage, using the Illumina iSeq platform. Low-quality reads were excluded based on predefined length (<80 bp) and quality thresholds (average Phred quality score <Q30). Taxonomic assignment was performed using EzBioCloud and reads were clustered into operational taxonomic units (OTUs) at 97% similarity using USEARCH (https://www.drive5.com/usearch/) and the UPARSE pipeline.

### Microbial profiling

Bacterial genera showing differential abundance between patients with GC and HCs were identified using R software, version 4.1.1. Alpha and beta diversity, as well as weighted and unweighted UniFrac distances, were calculated based on genus-level absolute abundance data using the ‘Phyloseq’ package in R. Statistical significance between GC and HC, as well as taxon composition profiles were analysed in R based on abundance quartile values, Wilcoxon rank-sum test, and fold-change (FC). R was also used to perform univariate logistic regression. The quartiles for each taxon were determined based on their distribution among HCs. If any quartile had a value of zero, it was categorised into quantiles or as zero/non-zero where applicable. Odds ratios and 95% confidence intervals (CIs) were calculated for all logistic regressions. Differential abundance analysis was performed using the Linear discriminant analysis Effect Size (LEfSe) method, and a heatmap and cladogram for taxa at the genus level were generated using the ‘microbiomeMarker’ package in R.

### Functional profiling

The PICRUSt1/2 algorithm within the EzBioCloud 16S-based microbiome taxonomic profiling pipeline was used to create functional profiles. Raw sequence reads were obtained by applying default parameters and identifying relevant reads in the reference database [25]. Functional profiles inferred from the oral microbiome were annotated using the Kyoto Encyclopaedia of Genes and Genomes (KEGG) ortholog and pathway modules. Accordingly, the vector of gene numbers for each OTU was multiplied by its abundance in every sample. The accuracy of each functional profile was assessed using the nearest-sequenced taxon index.

### Protein quantification by enzyme-linked immunosorbent assay (ELISA)

Cytokine and chemokine levels in plasma samples were quantified by ELISA using commercial kits and the Bio-Plex System (Bio-Rad, Hercules, CA, USA), following the manufacturer’s specifications. Kits for the growth differentiation factor 15 (#BMS2258) and transforming growth factor beta 1 (TGF-β1) (#BMS249-4) were purchased from Life Technologies; those for the nuclear factor kappa-light-chain-enhancer of activated B cells (NF-κB) (#CSB-E12107h) was purchased from CUSABIO (Houston, TX, USA); and those for the CX3C motif chemokine receptor 1 (#MBS2505819), *p*-Smad2 (#MBS269933), and *p*-Smad3 (#MBS269936) were purchased from MyBioSource (San Diego, CA, USA). To examine potential host–microbe associations, plasma markers related to the phosphoinositide 3-kinase/protein kinase B, such as serine/threonine protein kinase/nuclear factor kappa-light-chain-enhancer of activated B (PI3K/AKT/NF-κB), interleukin-6/Janus kinase/signal transducer and activator of transcription 3 (IL-6/JAK/STAT3), and transforming growth factor-β/SMAD family member 2/3/4 (TGF-β/Smad2/3/4) pathways were measured and matched with the corresponding saliva sequencing samples. Associations between relative microbial abundance and protein expression were evaluated by Spearman correlation.

### Construction of a machine learning model for classifying GC

Machine learning models were implemented using Python 3.10. A repeated stratified 5-fold cross-validation (10 repetitions) approach was applied to randomise GC and HC samples into training and test datasets, ensuring balanced class proportions for internal validation. The independent validation cohort was used exclusively for external performance evaluation to prevent data leakage. Only genera with ≥10 total counts across all samples were retained for analysis. Gradient Boosting Machine, Light Gradient Boosting Machine, Random Forest, and eXtreme Gradient Boosting (XGBoost) algorithms were used to classify GC *vs*. HC samples based on genus-level taxonomic profiles. Hyperparameter settings and additional implementation details for each algorithm are summarised in Table S1 to ensure reproducibility. Model performance was assessed using multiple metrics, including the area under the Receiver-Operating Characteristic curve (AUC), sensitivity, specificity, precision, recall, F1-score, and confusion matrices. During preliminary optimisation, oversampling using the Borderline-1 SMOTE algorithm was explored but not adopted in the final analysis, as it did not improve performance stability. To compare classification performance across algorithms, all models were trained and evaluated using the same cross-validation framework described above.

### Statistical analysis

Following quality filtering, the *16S* rRNA sequencing data were subjected to taxonomic and functional profiling with the significance level set to 0.05. The demographic and clinical characteristics of the HC and GC groups were compared using *t*-tests for continuous variables (*e.g.* age and BMI) and chi-square tests for categorical variables (*e.g.* sex, smoking, and alcohol consumption). Associations between oral microbiota composition and GC were assessed using univariate and multivariate logistic regression models adjusted for age, sex, BMI, smoking status, and alcohol consumption to prevent potential confounding factors. ROC curves were used to evaluate the diagnostic and predictive potential of oral microbiota for GC. For multiple testing correction, *p*-values from Wilcoxon rank-sum and regression analyses were adjusted using the Benjamini–Hochberg false discovery rate (FDR) method. LEfSe analysis was performed under default parameters (*α* = 0.05 for the Kruskal–Wallis test, LDA score > 2.0) using the Galaxy platform. All statistical analyses were conducted using R v4.3.2, Python v3.12.1, and SPSS v29.0, and the results were visualised using the MultiExperiment Viewer v4.9.0.

## Results

### Clinical and diagnostic characteristics

The demographic and clinical characteristics of the study population are summarised in Table 1. The discovery set included 77 patients with GC and 477 HCs, and the internal validation set included 36 patients with GC and 190 HCs. In both datasets, patients with GC were older and included a higher proportion of females compared with HCs. In the discovery set, BMI was comparable between the two groups; whereas in the validation set, the GC group had more individuals with a higher BMI. Non-smokers were prevalent in the HC group, whereas there were no differences in drinking habits. *H. pylori* was detected in approximately 70% of patients with GC screened (*n* = 107). Approximately 50–60% of patients tested negative for all three serum tumour markers (CEA, CA19-9, and CA72-4).

### Oral microbial composition is similar between GC and HC

**Figure 1.**
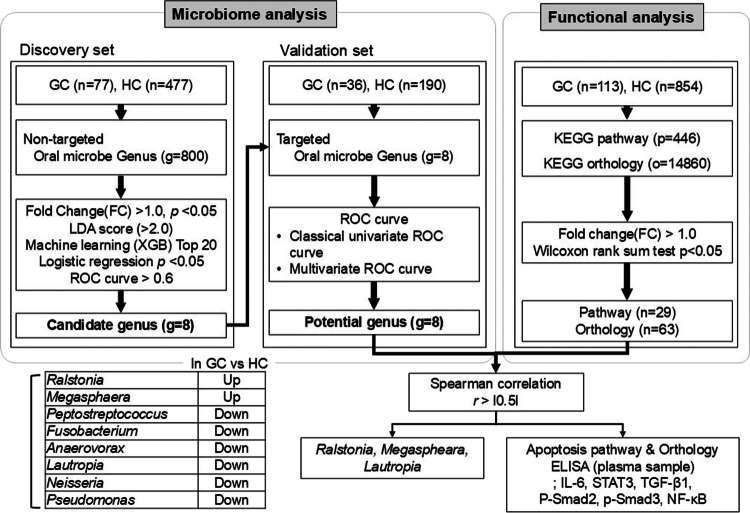
Flowchart of the study methodology.

**Figure 2.**
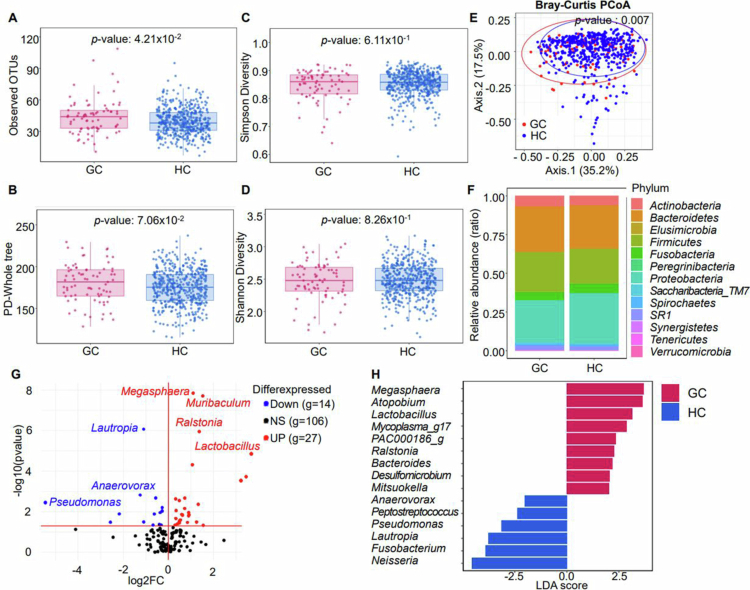
Differentially abundant bacterial taxa between GC and HCs. (A) Observed OTUs showing the number of different microorganisms, without considering their specific proportions or distribution. (B) Whole-tree phylogenetic diversity graph representing alpha diversity based on phylogeny. (C, D) Simpson and Shannon boxplot graphs representing microbiome evenness. (E) Bray–Curtis representation of beta diversity. (F) Phylum-level relative abundance of the 13 most common bacteria in the two groups. (G) Volcano plot showing differential abundance at the genus level in patients with GC compared with HCs. The plot is given as the log2 FC *vs*. the adjusted *p*-value obtained by the Wilcoxon rank sum test. More or less abundant genera were determined by *p* ≤ 0.05 and FC > 1 or < 1, respectively. (H) LEfSe analysis showing the differential enrichment of taxa at genus level (LDA > 2.0, *p* < 0.01). Red and blue denote genera significantly enriched in GC and HC, respectively. *p*-values from Wilcoxon rank-sum tests were adjusted using the Benjamini–Hochberg false discovery rate method. LEfSe analysis was performed using *α* = 0.05 for the Kruskal–Wallis test and an LDA threshold of 2.0.

To identify GC markers in the oral microbiome, we analysed the microbial composition in saliva samples, in which 800 genera were identified (Figure 1). Although the number of observed OTUs was higher in the GC group, no significant differences in whole-tree phylogenetic diversity, Simpson, or Shannon diversity indices were detected (Figure 2A–D). Evaluation of beta diversity using Bray–Curtis principal component analysis revealed overlap between the oral microbial communities of the two groups (Figure 2E). These findings suggest similar overall microbial diversity and community structure, with only subtle compositional differences between GC and HCs.

### Differential enrichment of the oral microbiota in GC and HC samples

To identify significant differences in relative abundance of microorganisms among GC and HC samples, we compared single genera (Figure 2F). The analysis revealed 27 genera, including *Megasphaera*, *Muribaculum*, and *Ralstonia*, that were more abundant in GC, whereas 14 genera, including *Lautropia*, were less abundant in GC (Figure 2G, Table S2). LEfSe analysis (LDA > 2.0, *p* < 0.05) identified nine genera enriched in GC, including *Megasphaera* and *Ralstonia*, and six enriched in HCs, such as *Neisseria* and *Lautropia* (Figure 2H). These findings highlight the differential abundance of *Megasphaera*, *Ralstonia*, and *Lautropia* in GC.

### High-performance machine learning model based on oral microbial compositional differences

**Figure 3.**
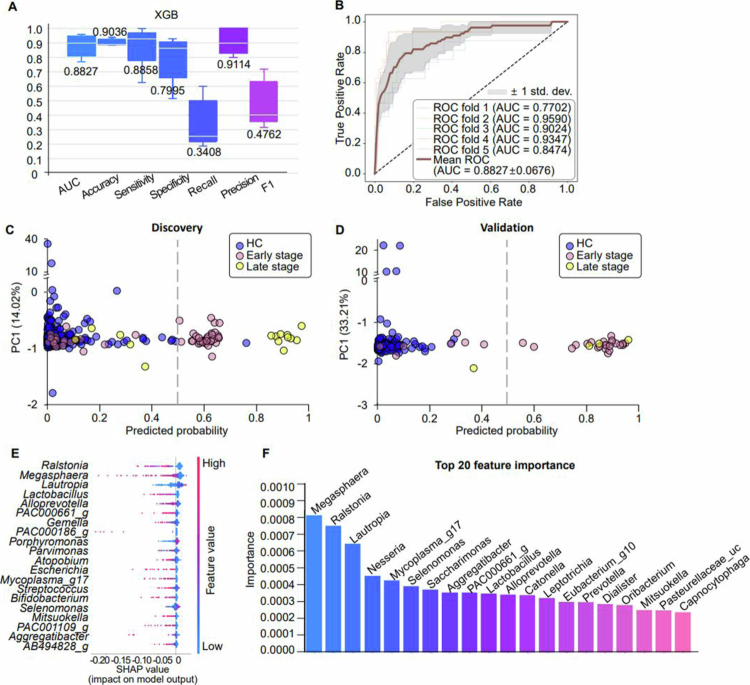
GC prediction model. After removing rare taxa, three models were constructed using the remaining 147 genera. (A) Performance of the XGBoost model. (B) ROC curve of the XGBoost model. (C, D) Performance of the XGBoost model in distinguishing early-stage (stage I, pink) and advanced-stage (stages II–IV, yellow) patients with GC from HCs (blue) in the (C) discovery and (D) validation datasets. (E) Key values obtained by combining various features to determine the importance of one of them and averaging the change depending on the variable’s presence or absence. (F) Variable importance histogram of the best model.

Comparison of multiple algorithms showed the highest overall performance for the XGBoost classifier (Figure 3A, Table S3). Specifically, the XGBoost model without oversampling achieved an AUC of 0.8827, when discriminating patients with GC from HCs (Figure 3B, Figures S1–S5). Additionally, the model achieved AUCs of 0.827 and 0.832 for detecting early and late GC stages in the discovery set and 0.871 and 0.875 in the validation set, respectively, while applying a threshold of 0.5 (Figure 3C,D), therefore supporting its potential as a stage-independent, non-invasive diagnostic tool for GC. To identify key genera associated with GC, we calculated importance scores or SHAP values from the XGBoost model (Figure 3E,F), obtaining 20 possible candidates.

### Association between high-ranking oral microbial genera and GC risk

To evaluate the GC risk associated with high-ranking genera from the XGBoost model, we performed univariate and multivariate logistic regression analyses (Table S4). Genera with high importance scores in the model demonstrated significant associations with GC. Specifically, *Aggregatibacter*, *Megasphaera*, and *Ralstonia* were positively associated with GC risk, whereas *Lautropia* and *Oribacterium* showed inverse associations.

### Identification and validation of potential microbial markers for GC diagnosis

**Figure 4.**
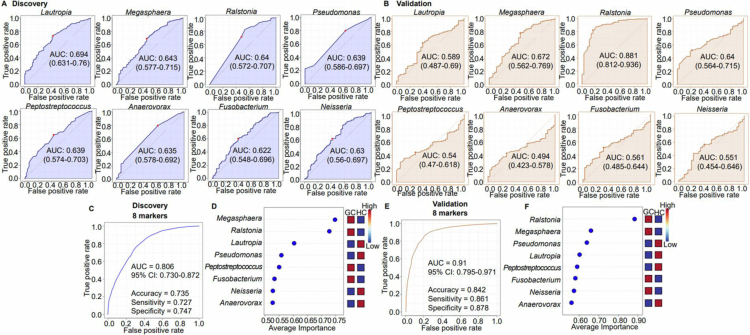
ROC curve for the diagnostic and predictive potential of oral microbiota in GC. (A) Discovery set analysis using microbiome data and machine learning-based methodology. (B) ROC curve computed based on the eight genera identified in the discovery set. (C) Average importance score of the genera from the discovery set. (D) Validation set analysis using microbiome data and machine learning-based methodology. (E) ROC curve computed based on the eight genera identified in the validation set. (F) Average importance score of the genera from the validation set.

The diagnostic utility of oral microbial profiling was evaluated by ROC analysis. Eight genera, including *Lautropia*, *Megasphaera*, *Ralstonia*, *Pseudomonas*, *Peptostreptococcus*, *Anaerovorax*, *Fusobacterium*, and *Neisseria*, presented individual AUC values of >0.6 (Figure 4A,B). A combined multivariate model incorporating these genera yielded AUCs of 0.806 and 0.91 in the discovery and validation datasets, respectively, indicating strong diagnostic accuracy (Figure 4C–F). Notably, *Lautropia*, *Megasphaera*, and *Ralstonia* showed robust performance, with AUCs of 0.752 and 0.862 in the respective datasets (Figure S6). To assess stage-specific relevance, we compared the relative abundances of the above-indicated eight genera across HCs and early- and late-stage GCs (Figure S7). *Anaerovorax*, *Peptostreptococcus*, and *Pseudomonas* showed differential abundance between HC and early-stage GC, whereas *Lautropia*, *Megasphaera*, and *Ralstonia* exhibited significant differences across all three groups.

### Influence of *H. pylori* infection and tumour status on microbial marker abundance in patients with GC

To assess whether the microbial marker abundance was influenced by *H. pylori* infection, we compared the relative abundances of the selected microbial genera in *H. pylori*-positive (*n* = 74) and -negative (*n* = 33) patients with GC. Despite the lack of group-level differences (Table S5), the eight-genera model achieved an AUC of 0.711 when discriminating *H. pylori*-negative patients with GC from HCs (Figure S8). To assess the diagnostic relevance of oral microbial markers in the absence of conventional tumour indicators, patients with GC were stratified by tumour marker status. *Peptostreptococcus* was the only genus with significantly higher abundance in marker-positive patients (Table S6). Notably, the microbial marker model maintained high diagnostic performance in marker-negative patients with GC *vs*. HCs, with AUCs of 0.806 (sensitivity: 0.7800; specificity: 0.7652) and 0.890 (sensitivity: 0.8947, specificity: 0.8474) in the discovery and validation datasets, respectively (Figure S8).

### Functional associations of *Ralstonia* and *Lautropia* with GC-related signalling

**Figure 5.**
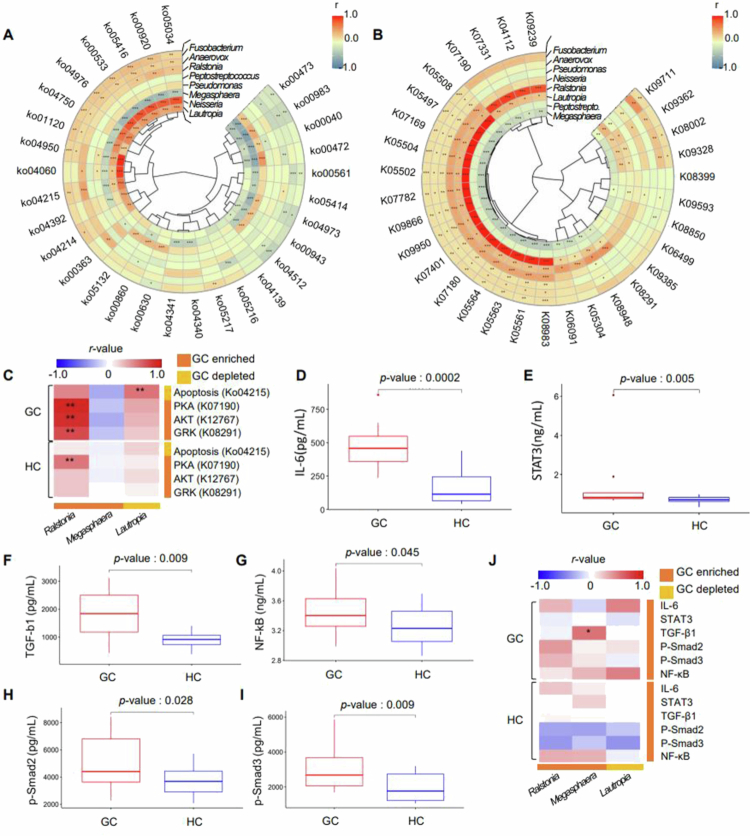
Expression of apoptosis-related genes in GC and HCs. (A) Heatmap of the Spearman’s rank correlation analysis between 8 genera and 30 significant pathways in GC. (B) Heatmap of the Spearman’s rank correlation analysis between 8 genera and 63 significant orthologs in GC. (C) Correlation analysis between 3 genera (columns) and 4 functional profiles (Ko04215-apoptosis, K07190-phosphorylase kinase alpha/beta subunit, K12767-serine/threonine-protein kinase, K08291-G protein-coupled receptor kinase) in GC and HCs. Full pathway and ortholog names are provided in Table S8. (D–I) Plasma level of (D) IL-6, (E) STAT3, (F) TGF-*β*, (G) NF-kB, (H) *p*-Smad2, and (I) *p*-Smad3 from 16 samples selected based on age and sex variance in each group. (J) Correlation analysis between 3 genera (columns) and 6 target molecules (rows) in GC and HCs. *p < 0.05, **p < 0.01, and ***p < 0.001. PKA; phosphorylase kinase alpha/beta subunit, AKT; protein kinase B, GRK; G protein-coupled receptor kinases, IL-6; Interleukin-6, STAT3; Signal transducer and activator of transcription 3, TGF-β1; Transforming growth factor beta 1, NF-κB; Nuclear factor kappa-light-chain-enhancer of activated B cells. All *p*-values were adjusted for multiple comparisons using the Benjamini–Hochberg FDR correction.

To explore the functional relevance of the eight candidate genera, we performed KEGG pathway and ortholog analyses. Significant differences were detected across 30 pathways and 63 orthologs (Figure S9 and Table S7). Correlation analysis revealed notable associations of *Lautropia* and *Megasphaera* with apoptosis (ko04215), cytokine interaction (ko04060), and inflammatory mediator regulation of TRP channels (ko04750) (Figure 5A, Figure S10, and Table S6). Interestingly, *Ralstonia* and *Lautropia* exhibited opposite correlations with key pathways and orthologs. *Ralstonia* correlated positively with G-protein-coupled receptor kinase (GRK) (K08291), phosphorylase kinase beta (K07190), and serine/threonine protein kinase (K12767) (Figure 5B,C). *Ralstonia* and *Lautropia* may be associated with altered apoptotic signalling in gastric cancer.

### *Megasphaera* may promote GC by modulating TGF-β1 signalling

Levels of NF-κB, IL-6, TGF-β1, *p*-Smad2, and *p*-Smad3 were significantly elevated in GC (Figure 5D–I). Among the identified genera, *Megasphaera* showed a positive correlation with TGF-β1 expression (Figure 5J), suggesting a possible role in GC-related TGF-β/Smad pathway activation.

## Discussion

Our findings highlight the possible association between specific oral microbial genera and GC, as well as their role in promoting carcinogenesis-related pathways. Using *16S* rRNA sequencing, we compared the oral microbiota of patients with GC and HCs. Despite similar overall microbial diversity and community structure, patients with GC exhibited greater taxonomic richness. The genera *Lautropia*, *Megasphaera*, *Ralstonia*, *Pseudomonas*, *Peptostreptococcus*, *Anaerovorax*, and *Neisseria* were identified as potential diagnostic markers. *Megasphaera* and *Ralstonia* were associated with increased GC risk, whereas *Lautropia* showed an inverse relationship. Moreover, these genera appeared to be related to pathways associated with apoptosis, cellular proliferation, and carcinogenesis, suggesting possible involvement in GC pathogenesis.

Differences in gut, gastric, and faecal microbiota between patients with GC and HCs have been extensively documented [10,26,27]. However, despite the suggested transfer of oral microbes to the gastrointestinal tract [13], their role in GC remains underexplored. Seven studies have examined oral microbiota using saliva, tongue coating, or mucosal swabs [6,13,18–23]; but only three assessed diagnostic accuracy (AUC ≈ 0.600–0.824) [6,18,19]. Compared with these studies, our model achieved an AUC of 0.91 in the internal validation cohort, indicating superior diagnostic performance based on saliva microbiota data. Although approximately 40 microbial genera were previously suggested to be potentially related to GC risk [6,13,18–23], independent validation or mechanistic investigation were still warranted. Only two studies addressed microbial function, either across multiple cancers [22] or by reporting limited associations with inflammatory pathways via shotgun metagenomics [23]. In contrast, our study identified and validated GC-associated oral markers and demonstrated their functional relevance in gastric carcinogenesis via cytokine profiling.

*Lautropia* is commonly associated with periodontal disease [28]. In this study, its low abundance in patients with GC correlated positively with apoptotic signalling, suggesting a potential protective role. This contrasts with findings by Xu et al., who reported increased *Lautropia* in the tongue coating of patients with GC [19], but agrees with the findings of Chen et al., who reported *Lautropia* depletion in the oral cavity of patients with oesophageal squamous cell carcinoma [29]. Notably, neither study explored its functional implications. Baraniya et al. showed that *Lautropia* enrichment in specific oral squamous cell carcinoma subtypes was linked to downregulation of CD36, a tumour-related factor [30]. Together, these findings suggest that *Lautropia* depletion may play a possible role in GC progression through altered apoptotic pathways.

Previous studies have reported decreased *Megasphaera* abundance in the oral cavity of patients with GC, which contrasts with our findings [27]. This difference may be attributable to distinct sampling sites, as our study analysed unstimulated whole saliva, which represents a more comprehensive microbial niche. However, *Megasphaera* is commonly enriched in the gut and faeces, and its relevance to GC has been noted before [31]. Our data suggest that *Megasphaera* levels correlate with plasma TGF-β1, a cytokine known to promote carcinogenesis. Indeed, TGF-β1 signalling, mediated via Smad2/3 phosphorylation, regulates transcriptional programmes involved in GC cell migration and invasion [32,33]. The elevated levels of *p*-Smad2 and *p*-Smad3 in patients with GC compared with HCs further support the hypothesis that *Megasphaera* may be involved in GC-related TGF-β/Smad2/3 signalling.

**Figure 6.**
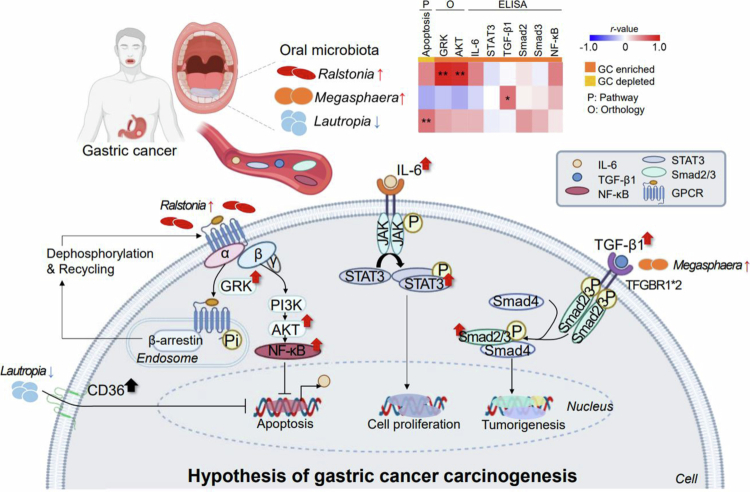
Proposed mechanism of gastric carcinogenesis illustrating the directionality of molecular differences observed in this study. The schematic framework is adapted from previously reported carcinogenic signalling pathways in gastric cancer, including the TGF-β/Smad2/3 pathway [32,33], the PI3K/AKT/NF-κB pathway [38–41], and the IL-6–mediated JAK/STAT3 pathway [42,43]. Molecules analysed in this study are highlighted, with red and blue arrows indicating relatively higher and lower levels, respectively, in patients with GC compared with HCs, based on group-wise comparisons of PICRUSt-inferred orthologs and plasma cytokine measurements. The figure is intended to contextualise these observational findings within established literature-based signalling frameworks and does not represent direct experimental validation of mechanistic pathways. TGF-β; transforming growth factor beta, TFGBR; TGF-*β* receptor, PI3K; phosphoinositide 3-kinases, AKT; protein kinase B, GPCR; G protein-coupled receptor, GRK; G protein-coupled receptor kinases, NF-κB; nuclear factor kappa-light-chain-enhancer of activated B cells, IL-6; interleukin 6, STAT3; signal transducer and activator of transcription 3, JAK; Janus kinase, PTEN; phosphatase and tensin homologue.

To our knowledge, our study is the first to report increased *Ralstonia* abundance in the oral cavity of patients with GC. *Ralstonia*, a gram-negative bacterium of the *Burkholderiaceae* family, has typically low pathogenicity but is frequently associated with chronic inflammation and mucosal infections [34]. Consistent with our findings, elevated *Ralstonia* levels have been reported in the gastric mucosa of patients with early-stage GC than in those with superficial gastritis [35]. Its known pro-inflammatory potential [36] may explain enrichment in GC. Functional analysis suggested that *Ralstonia* abundance may be associated with higher levels of GRK and PI3K orthologs. Given that GRK and AKT are involved in inflammatory and apoptotic pathways [37], these associations may reflect a potential link between oral microbial dysbiosis and altered host signalling in GC. Activation of the G protein-coupled receptor upon ligand binding initiates downstream cascades including the PI3K/AKT/NF-κB pathway, which promotes tumour growth and inhibits apoptosis [38–40]. Microbial imbalance, including abundant *Ralstonia*, has been shown to activate NF-κB signalling, which may be associated with GC progression [41]. Activated NF-κB enhances transcription of IL-6, which engages the JAK/STAT3 pathway through receptor binding and phosphorylation [42]. Nuclear STAT3 then drives expression of genes that support tumour cell proliferation and survival [43]. The observed elevated levels of IL-6 and STAT3 in the blood of patients with GC support the potential role of *Ralstonia* in modulating these oncogenic pathways. A schematic representation of the proposed mechanism is shown in Figure 6. Nonetheless, these functional associations should be interpreted with caution, as they are inferred from PICRUSt-based KEGG predictions derived from *16S* sequencing data and do not represent direct functional activity.

In the present study, *Peptostreptococcus* and *Fusobacterium* were more abundant in the oral microbiota of patients with GC than in HCs, whereas *Pseudomonas*, *Anaerovorax*, and *Neisseria* showed the opposite trend. These findings align with previous results. Wu et al. reported increased *Peptostreptococcus* in saliva from patients with GC (odds ratio: 1.56; 95% confidence interval: 1.16–2.10) [23]. *P. stomatis* was also elevated in gastric biopsies of patients with GC, potentially facilitating colonisation under hypoxic and acidic tumour conditions [44,45]. The enrichment of *Fusobacterium* in GC is consistent with prior reports [6,20]. Although *Fusobacterium nucleatum* is a virulent anaerobe linked to oral and colorectal cancers [46], herein, it did not differ significantly between GC and HCs, suggesting other *Fusobacterium* species may be more relevant in GC pathogenesis.

Our findings also corroborate those of Wu et al. reporting reduced *Pseudomonas* abundance in GC [18]. *Pseudomonas aeruginosa* produces azurin, a cupredoxin with pro-apoptotic effects [47]; therefore, its low abundance may hamper apoptotic signalling in GC.

*Anaerovorax* was also scarce in patients with GC and, to our knowledge, this is the first report suggesting its potential association with GC risk. Notably, a similar trend has been observed in the faecal microbiota of patients with irritable bowel syndrome [48].

Several studies have linked a high GC risk to low *Neisseria* levels in the oral cavity [6,18], which is consistent with our findings. *Neisseria* comprises both commensal and pathogenic species: *N. lactamica* is associated with bactericidal activity against *N. meningitidis* [49]; whereas *N. gonorrhoeae* is a major pathogen [50,51]. Some *Neisseria* species produce high levels of acetaldehyde, particularly in the presence of alcohol, which may contribute to carcinogenesis [13]. Collectively, *Peptostreptococcus, Fusobacterium, Neisseria,* and *Pseudomonas* may contribute to gastric carcinogenesis via distinct microbial-host interactions, warranting further investigation as candidate diagnostic or prognostic markers.

To further evaluate the diagnostic performance of our model in clinically relevant subgroups, we conducted analyses based on cancer stage, *H. pylori* infection, and tumour marker status. In early-stage GC, the classifier achieved an AUC of 0.871 in the validation set. A large-scale study of over 5,000 individuals reported an AUC of 0.848 for a serum-based 12-miRNA panel, which increased to 0.884 when clinical variables such as age and *H. pylori* serology were included [52]. Notably, our oral microbiota–based model achieved comparable performance without invasive sampling or clinical inputs. *Lautropia*, *Megasphaera*, *Ralstonia*, *Anaerovorax*, *Peptostreptococcus*, and *Pseudomonas* were altered in early-stage GC, highlighting how microbial shifts occur early in carcinogenesis. *H. pylori* infection had little effect on marker abundance and diagnostic performance in *H. pylori*–negative patients (AUC = 0.711). Conventional tumour markers such as CEA, CA19-9, and CA72-4 have limited sensitivity, especially when they all fall within the reference range. A prior study reported sensitivities of 0.165, 0.217, and 0.317 for CEA, CA19-9, and CA72-4, respectively, and only 0.428 when all three were combined [53]. In contrast, our model achieved a sensitivity of 0.8947 in patients negative for all three markers, demonstrating its value in cases missed by conventional biomarkers.

This study benefits from robust methodological and analytical approaches. The relatively large, well-defined cohort allowed for a discovery-validation framework, which boosted reproducibility. Importantly, microbial biomarkers were functionally validated through pathway enrichment and cytokine profiling, linking taxonomic shifts to tumour-relevant processes. The model achieved strong diagnostic performance (AUC = 0.91) in the validation set, surpassing previously reported values in oral microbiome-based GC studies. These results suggest the clinical potential of oral microbial profiling for early, non-invasive detection of GC.

Nevertheless, some limitations of the study should be noted. The sample size imbalance between GC and HC groups may have introduced statistical bias. As all participants were from the Korean population, generalisability to other ethnic or geographic groups is limited. Although all saliva samples were collected under standardised fasting and clinical protocols, information on oral health status, recent antibiotic use, and detailed dietary intake was not available. These unmeasured factors may partially influence oral microbial composition and should be addressed in future prospective studies. Sex-related variation in oral microbiota has been previously reported [54] and was not directly addressed in the present study; nevertheless, our main findings remained robust after adjustment for sex and other demographic variables. The observed correlations between oral microbial taxa and pathways require further validation through metatranscriptomic or *in-vitro* functional studies. Lastly, *16S* rRNA sequencing should be complemented by shotgun metagenomics to achieve higher taxonomic and functional resolution [55].

## Conclusions

We identified key oral microbial genera—particularly *Ralstonia*, *Megasphaera*, and *Lautropia*—which are significantly associated with GC and associated with pathways related to carcinogenic signalling. By integrating taxonomic, functional, and cytokine-level evidence, our study provides novel insights into microbe-driven mechanisms of gastric carcinogenesis. Notably, the proposed classification method was effective for identifying early-stage and tumour-marker–negative GCs, highlighting its utility in diagnostically challenging subgroups. These findings support the potential of oral microbiota as markers for biologically informed, non-invasive screening and risk stratification in GC.

## Supplementary Material

Supplementary material.docx Supplementary material.docx

## Data Availability

The raw *16S* rRNA sequencing data have been deposited in the NCBI Sequence Read Archive (SRA) under BioProject accession number PRJNA1302131 and are publicly available.

## Patent

This research resulted in the registration and filing of the following patents: Biomarker for diagnosing gastric cancer and use thereof (Patent No. 10-2025-0061698).
