# Supplementary material for: Diagnostic oral microbiota signatures for gastric cancer and associations with carcinogenic signaling pathways
Source: J Oral Microbiol. 2026 Jan 11;18(1):2613531. doi: 10.1080/20002297.2026.2613531 (PMC12794691; doi:10.1080/20002297.2026.2613531)
Supplement: Supplementary material.docx [file ZJOM_A_2613531_SM3518.docx]

**Table S1**. Hyperparameter settings for machine learning algorithms used in this study.

| **Algorithm** | **n_estimators** | **Learning rate** | **Max depth** | **Num leaves** | **Boosting type** |
| --- | --- | --- | --- | --- | --- |
| XGBoost | 100 | 0.1 | 50 | - | - |
| LightGBM | 1000 | - | - | 30 | Goss |
| Random Forest | 100 | - | 50 | - | - |
| Gradient Boosting | - | 0.13 | 100 | - | - |

**Table S2**. Relative abundances of genus level microbiome after removing rare taxa

| **Genus** | **GC** | **HC** | **p-value^a^** | **Up/down** |
| --- | --- | --- | --- | --- |
| *Megasphaera* | 0.9114±0.9361 | 0.4230±0.5399 | 1.36E-08 | Up |
| *Lautropia* | 0.5199±1.0427 | 1.1063±1.6791 | 8.31E-07 | Down |
| *Ralstonia* | 0.0279±0.0429 | 0.0096±0.0218 | 1.12E-06 | Up |
| *Lactobacillus* | 0.1379±0.5519 | 0.0109±0.0486 | 1.39E-05 | Up |
| *Bifidobacterium* | 0.0107±0.0238 | 0.0040±0.0119 | 4.79E-05 | Up |
| *Atopobium* | 1.2603±1.4098 | 0.7970±1.2444 | 0.0002 | Up |
| *Mycoplasma_17* | 0.0801±0.4455 | 0.0075±0.0836 | 0.0003 | Up |
| *Anaerovorax* | 0.0058±0.0199 | 0.0164±0.0576 | 0.0019 | Down |
| *Peptostreptococcus* | 0.0457±0.0685 | 0.0669±0.0809 | 0.0020 | Down |
| *Desulfomicrobium* | 0.0271±0.0608 | 0.0156±0.054 | 0.0021 | Up |
| *Escherichia* | 0.0047±0.0177 | 0.0028±0.0448 | 0.0028 | Up |
| *Bacteroides* | 0.0443±0.0748 | 0.0304±0.1457 | 0.0029 | Up |
| *Pseudomonas* | 0.002±0.0053 | 0.1521±0.4107 | 0.0038 | Down |
| *Paraburkholderia* | 0.0023±0.0059 | 0.0009±0.0038 | 0.0041 | Up |
| *Mitsuokella* | 0.0149±0.0607 | 0.0048±0.0169 | 0.0044 | Up |
| *Propionivibrio* | 0.0007±0.0037 | 0.0026±0.0071 | 0.0048 | Down |
| *Fusobacterium* | 3.7921±2.5118 | 4.5884±2.8411 | 0.0064 | Down |
| *Eubacterium_14* | 0.0132±0.0320 | 0.0073±0.0216 | 0.0070 | Up |
| *GU304534* | 0.0041±0.0184 | 0.0027±0.0251 | 0.0088 | Up |
| *Neisseria* | 15.356±12.115 | 18.581±11.262 | 0.0098 | Down |
| *JN713301* | 0.0039±0.0180 | 0.0011±0.0064 | 0.0109 | Up |
| *Catonella* | 0.0890±0.1409 | 0.1137±0.1302 | 0.0110 | Down |
| *Veillonellaceae_uc* | 0.0003±0.0016 | 0.0082±0.0339 | 0.0125 | Down |
| *Oribacterium* | 0.2619±0.2225 | 0.4007±0.4321 | 0.0132 | Down |
| *Ruminococcus* | 0.0014±0.0039 | 0.0008±0.0037 | 0.0135 | Up |
| *PAC000661* | 0.1700±0.1995 | 0.1124±0.1522 | 0.0135 | Up |
| *Alloprevotella* | 2.9335±2.3013 | 2.3414±2.0714 | 0.0220 | Up |
| *AB494828* | 0.0206±0.0421 | 0.0136±0.0343 | 0.0274 | Up |
| *Spirochaetaceae_uc* | 0.0408±0.1269 | 0.0286±0.1459 | 0.0307 | Up |
| *Flexilinea* | 0.0006±0.0034 | 0.0018±0.0061 | 0.0314 | Down |
| *Neisseriaceae_uc* | 0.0788±0.1166 | 0.1685±0.4433 | 0.0320 | Down |
| *Prevotellaceae_uc* | 0.0014±0.0048 | 0.0176±0.0634 | 0.0331 | Down |
| *Roseburia* | 0.0026±0.0072 | 0.0016±0.0075 | 0.0332 | Up |
| *Oscillibacter* | 0.0069±0.0240 | 0.0018±0.0092 | 0.0333 | Up |
| *Sneathia* | 0.2545±0.9284 | 0.1806±0.8929 | 0.0365 | Up |
| *PAC001141* | 0.0629±0.1463 | 0.0477±0.1467 | 0.0396 | Up |
| *Cardiobacterium* | 0.1272±0.1426 | 0.1684±0.1976 | 0.0419 | Down |
| *Porphyromonas* | 4.1801±3.4874 | 5.1829±4.1376 | 0.0437 | Down |
| *Streptococcus* | 7.1464±5.7735 | 5.8768±5.6669 | 0.0441 | Up |
| *Ottowia* | 0.0510±0.1064 | 0.0810±0.1574 | 0.0461 | Down |
| *Scardovia* | 0.0223±0.0752 | 0.0064±0.0410 | 0.0473 | Up |
| *Actinomyces* | 1.6281±1.3189 | 1.4139±1.3351 | 0.0624 | Up |
| *Actinobaculum* | 0.0380±0.0740 | 0.0495±0.0801 | 0.0696 | Down |
| *Corynebacterium* | 0.2437±0.2498 | 0.3153±0.3220 | 0.0707 | Down |
| *Stenotrophomonas* | 0.0001±0.0012 | 0.0020±0.0093 | 0.0709 | Down |
| *DQ413083_f_uc* | 0.0001±0.0011 | 0.0345±0.4359 | 0.0759 | Down |
| *FJ470493* | 0.0061±0.0173 | 0.0036±0.0177 | 0.0786 | Up |
| *Brachymonas* | 0.0007±0.0031 | 0.0025±0.0146 | 0.0805 | Down |
| *PAC000677* | 0.1804±0.2466 | 0.1403±0.2420 | 0.0823 | Up |
| *Paludibacter* | 0.0498±0.0716 | 0.0701±0.1302 | 0.0839 | Down |
| *Aggregatibacter* | 0.6618±1.0094 | 0.4414±0.5871 | 0.0851 | Up |
| *Kingella* | 0.0819±0.1250 | 0.1150±0.2276 | 0.0911 | Down |
| *Haemophilus* | 7.7597±5.2558 | 8.7432±5.2523 | 0.0957 | Down |
| *Faecalibacterium* | 0.0054±0.0135 | 0.0030±0.0099 | 0.0966 | Up |
| *AF366267* | 0.0032±0.0106 | 0.0069±0.0239 | 0.0967 | Down |
| *Capnocytophaga* | 1.0626±0.9627 | 1.3074±1.3818 | 0.0969 | Down |
| *Aminicella* | 0.0316±0.0633 | 0.0214±0.0509 | 0.1017 | Up |
| *Mobiluncus* | 0.0064±0.0191 | 0.0046±0.0222 | 0.1044 | Up |
| *GQ422727* | 0.0100±0.0137 | 0.0163±0.0232 | 0.1066 | Down |
| *Ruminococcus_2* | 0.0024±0.0073 | 0.0015±0.0087 | 0.1069 | Up |
| *EF586032* | 0.0063±0.0141 | 0.0037±0.0150 | 0.1080 | Up |
| *PAC001236* | 0.0022±0.0048 | 0.0039±0.0071 | 0.1102 | Down |
| *JN713427* | 0.0172±0.0303 | 0.0138±0.0281 | 0.1119 | Up |
| *Alistipes* | 0.0108±0.0422 | 0.0054±0.0573 | 0.1126 | Up |
| *Abiotrophia* | 0.0248±0.0567 | 0.0220±0.0978 | 0.1171 | Up |
| *Veillonella* | 13.660±7.9659 | 12.150±7.2659 | 0.1179 | Up |
| *Bergeyella* | 0.1894±0.1685 | 0.2324±0.2581 | 0.1227 | Down |
| *Subdoligranulum* | 0.0016±0.0051 | 0.0007±0.0027 | 0.1351 | Up |
| *Desulfobulbus* | 0.0029±0.0103 | 0.0044±0.0122 | 0.1373 | Down |
| *Peptoanaerobacter* | 0.0230±0.0432 | 0.0454±0.1160 | 0.1432 | Down |
| *Leptotrichia* | 0.9248±0.6585 | 1.2491±1.2772 | 0.1483 | Down |
| *Prevotella* | 20.557±11.849 | 18.176±10.286 | 0.1494 | Up |
| *Dialister* | 0.1938±0.3019 | 0.1347±0.1676 | 0.1589 | Up |
| *Enterobacterales* | 0.0028±0.0065 | 0.0102±0.0567 | 0.1612 | Down |
| *Cryptobacterium* | 0.0067±0.0216 | 0.0021±0.0065 | 0.1663 | Up |
| *Odoribacter* | 0.0076±0.0206 | 0.0040±0.0178 | 0.1720 | Up |
| *Parabacteroides* | 0.0019±0.0069 | 0.0015±0.0092 | 0.1906 | Up |
| *Eubacterium_23* | 0.0022±0.0074 | 0.0013±0.0058 | 0.1947 | Up |
| *Eubacterium_11* | 0.0119±0.0209 | 0.0105±0.0196 | 0.2015 | Up |
| *KI259813* | 0.0034±0.0082 | 0.0029±0.0116 | 0.2054 | Up |
| *Campylobacter* | 1.7930±1.3574 | 2.0765±1.6088 | 0.2206 | Down |
| *AF125206* | 0.0115±0.0255 | 0.0233±0.0778 | 0.2224 | Down |
| *AM420052* | 0.0105±0.0182 | 0.0194±0.0456 | 0.2305 | Down |
| *Sphingomonas* | 0.0015±0.0052 | 0.0028±0.0106 | 0.2655 | Down |
| *Propionibacterium* | 0.0003±0.0016 | 0.0007±0.0031 | 0.2669 | Down |
| *Eubacterium_5* | 0.0016±0.0067 | 0.0005±0.0023 | 0.2682 | Up |
| *Shuttleworthia* | 0.0065±0.0201 | 0.0033±0.0089 | 0.2933 | Up |
| *Schwartzia* | 0.0102±0.0164 | 0.0144±0.0237 | 0.2952 | Down |
| *Treponema* | 0.9952±1.8664 | 0.7828±1.3110 | 0.3160 | Up |
| *PAC001109* | 0.0198±0.0536 | 0.0136±0.0631 | 0.3181 | Up |
| *Mycoplasma_4* | 0.1833±0.3015 | 0.1480±0.3410 | 0.3288 | Up |
| *PAC001341* | 0.0628±0.1254 | 0.0762±0.1786 | 0.3333 | Down |
| *Porphyromonadaceae_uc* | 0.0009±0.0049 | 0.0017±0.0081 | 0.3345 | Down |
| *Pyramidobacter* | 0.0036±0.0119 | 0.0024±0.0116 | 0.3488 | Up |
| *AF125205* | 0.0098±0.0491 | 0.0036±0.0183 | 0.3597 | Up |
| *Mannheimia* | 0.0597±0.0882 | 0.0778±0.2400 | 0.3667 | Down |
| *PAC001354* | 0.0045±0.0089 | 0.0038±0.0080 | 0.3676 | Up |
| *Filifactor* | 0.0692±0.1035 | 0.0897±0.2362 | 0.3818 | Down |
| *Mogibacterium* | 0.1113±0.1618 | 0.0884±0.0895 | 0.3846 | Up |
| *Tannerella* | 0.2500±0.2867 | 0.2856±0.3461 | 0.4013 | Down |
| *Alloscardovia* | 0.0163±0.0939 | 0.0039±0.0160 | 0.4201 | Up |
| *Blautia* | 0.0029±0.0090 | 0.0018±0.0062 | 0.4208 | Up |
| *PAC001057_uc* | 0.0053±0.0138 | 0.0119±0.0392 | 0.4268 | Down |
| *AM420198* | 0.0030±0.0062 | 0.0031±0.0091 | 0.4378 | Down |
| *Pasteurellaceae_uc* | 0.4754±0.8629 | 0.5208±1.0715 | 0.4380 | Down |
| *AM419981* | 0.0012±0.0042 | 0.0056±0.0377 | 0.4399 | Down |
| *Howardella* | 0.0016±0.0051 | 0.0011±0.0039 | 0.4402 | Up |
| *Lachnospiraceae_uc* | 0.0012±0.0038 | 0.0034±0.0237 | 0.4507 | Down |
| *Selenomonas* | 0.6152±0.6222 | 0.6243±0.5746 | 0.4541 | Down |
| *Lachnospira* | 0.0016±0.0054 | 0.0012±0.0060 | 0.4625 | Up |
| *Saccharimonas* | 0.8733±1.2181 | 0.7160±0.9285 | 0.4764 | Up |
| *Acinetobacter* | 0.0026±0.0156 | 0.0012±0.0063 | 0.5207 | Up |
| *Gemella* | 0.5629±0.6139 | 0.5220±0.6459 | 0.5258 | Up |
| *Actinobacillus* | 0.0152±0.0515 | 0.0195±0.0465 | 0.5363 | Down |
| *Eubacterium10* | 0.2701±0.3374 | 0.2533±0.2629 | 0.5367 | Up |
| *Staphylococcus* | 0.0046±0.032 | 0.0018±0.0081 | 0.5376 | Up |
| *Bergeriella* | 0.0022±0.0110 | 0.0034±0.0200 | 0.5431 | Down |
| *Rodentibacter* | 0.0110±0.0366 | 0.0055±0.0346 | 0.5590 | Up |
| *Simonsiella* | 0.0452±0.1223 | 0.0510±0.1017 | 0.5641 | Down |
| *JN713562* | 0.0538±0.2135 | 0.0534±0.2036 | 0.5903 | Up |
| *Slackia* | 0.0068±0.0155 | 0.0060±0.0145 | 0.5980 | Up |
| *Eggerthia* | 0.0007±0.0025 | 0.0012±0.0045 | 0.6443 | Down |
| *Fretibacterium* | 0.1277±0.2293 | 0.1225±0.2127 | 0.6824 | Up |
| *Lachnoanaerobaculum* | 0.1279±0.1266 | 0.1609±0.2120 | 0.6914 | Down |
| *Enterococcus* | 0.0135±0.0205 | 0.0170±0.0335 | 0.7072 | Down |
| *Phocaeicola* | 0.0078±0.0212 | 0.0062±0.0139 | 0.7100 | Up |
| *Moryella* | 0.1362±0.1732 | 0.1462±0.2269 | 0.7164 | Down |
| *DQ003626* | 0.0053±0.0133 | 0.0069±0.0217 | 0.7218 | Down |
| *Desulfovibrio* | 0.0053±0.0157 | 0.0044±0.0170 | 0.7524 | Up |
| *GU410548* | 2.9084±6.4281 | 2.5473±5.0640 | 0.7784 | Up |
| *AM420159* | 0.0291±0.0490 | 0.0547±0.1642 | 0.7928 | Down |
| *Streptobacillus* | 0.0038±0.0213 | 0.0021±0.0100 | 0.7989 | Up |
| *ASND* | 0.0334±0.0982 | 0.0349±0.1026 | 0.8008 | Down |
| *Rothia* | 3.5853±3.9923 | 3.7112±4.5231 | 0.8044 | Down |
| *Johnsonella* | 0.0059±0.0109 | 0.0075±0.0157 | 0.8418 | Down |
| *Olsenella* | 0.0125±0.0309 | 0.0087±0.0259 | 0.8509 | Up |
| *Bulleidia* | 0.0693±0.0556 | 0.0802±0.0930 | 0.8564 | Down |
| *Eikenella* | 0.0233±0.0349 | 0.0270±0.0416 | 0.9296 | Down |
| *Granulicatella* | 0.7755±0.7815 | 0.8526±1.1238 | 0.9341 | Down |
| *Butyrivibrio* | 0.0136±0.0178 | 0.0189±0.0367 | 0.9367 | Down |
| *Cardiobacteriaceae_uc* | 0.0031±0.0146 | 0.0027±0.0177 | 0.9516 | Up |
| *Parvimonas* | 0.2223±0.3076 | 0.2108±0.3056 | 0.9640 | Up |
| *Wolinella* | 0.0008±0.0037 | 0.0022±0.0156 | 0.9795 | Down |

a. Statistical differences were calculated by Wilcoxon rank sum test

**Table S3.** The performance of classifying models.

|  | LGBM^a^ | GBM^b^ | RF^c^ |
| --- | --- | --- | --- |
| AUC score | 0.8744±0.0731 | 0.6604±0.0597 | 0.7611±0.0799 |
| Accuracy | 0.9018±0.0219 | 0.8554±0.029 | 0.8679±0.0067 |
| Sensitivity | 0.7667±0.1262 | 0.4442±0.1437 | 0.6908±0.1878 |
| Specificity | 0.909±0.0602 | 0.9131±0.0663 | 0.8012±0.127 |
| Recall | 0.3642±0.1072 | 0.365±0.1517 | 0.0392±0.032 |
| Precision | 0.8187±0.1411 | 0.4582±0.1554 | 0.6±0.4899 |
| F1 score | 0.4999±0.1211 | 0.4004±0.1475 | 0.0735±0.0601 |

All values are shown mean±SD. a. Light Gradient Boosting Machine, b. Gradient Boosting Machine, c. Random forest

**Table S4.** Logistic analysis results of features in machine learning model with best performance

|  | | Number of subject^a^ | | |  | | | |
| --- | --- | --- | --- | --- | --- | --- | --- | --- |
| Genus |  | GC | HC | Univariate OR^b^ (95%CI) | | *P* value^d^ | Multivariate OR^c^ (95%CI) | *P* value |
| *Aggregatibacter* | Continuous | 77 | 483 | 1.48 (1.10~1.99) | | 0.0096 | 1.43 (1.03~1.98) | 0.0335 |
|  | Quartile4 | 23 | 120 | 1.67 (0.82~3.40) | | 0.1571 | 0.50 (0.25~1.02) | 0.0585 |
|  | Quartile3 | 18 | 119 | 1.32 (0.63~2.77) | | 0.4660 | 1.55 (0.68~3.51) | 0.2976 |
|  | Quartile2 | 22 | 122 | 1.57 (0.77~3.21) | | 0.2157 | 5.77 (2.90~11.50) | 6.28×10^-7^ |
|  | Quartile1 | 14 | 122 |  | |  |  |  |
| *Alloprevotella* | Continuous | 77 | 483 | 1.12 (1.02~1.24) | | 0.0244 | 1.13 (1.01~1.25) | 0.0285 |
|  | Quartile4 | 33 | 121 | 1.97 (1.04~3.73) | | 0.0364 | 0.48 (0.23~0.98) | 0.0453 |
|  | Quartile3 | 13 | 120 | 0.78 (0.36~1.68) | | 0.5324 | 1.55 (0.67~3.59) | 0.3067 |
|  | Quartile2 | 14 | 119 | 0.85 (0.40~1.80) | | 0.6741 | 5.93 (2.94~11.96) | 6.39×10^-7^ |
|  | Quartile1 | 17 | 123 |  | |  |  |  |
| *Anaerovorax* | Continuous | 77 | 483 | <10^-9^ (<10^-9^~10.63) | | 0.1054 | <10^-9^ (<10^-9^~45.01) | 0.1518 |
|  | ≥0 | 9 | 143 | 0.31 (0.15~0.65) | | 0.0017 | 6.32 (3.14~12.70) | 2.24×10^-7^ |
|  | <0 | 68 | 340 |  | |  |  |  |
| *Capnocytophaga* | Continuous | 77 | 483 | 0.83 (0.65~1.06) | | 0.1344 | 0.82 (0.65~1.04) | 0.1044 |
|  | Quartile4 | 19 | 120 | 0.66 (0.35~1.24) | | 0.1980 | 0.51 (0.25~1.03) | 0.0593 |
|  | Quartile3 | 16 | 115 | 0.58 (0.30~1.12) | | 0.1071 | 1.48 (0.65~3.37) | 0.3448 |
|  | Quartile2 | 13 | 127 | 0.43 (0.21~0.86) | | 0.0172 | 5.51 (2.77~10.94) | 1.14×10^-6^ |
|  | Quartile1 | 29 | 121 |  | |  |  |  |
| *Catonella* | Continuous | 77 | 483 | 0.14 (0.01~1.78) | | 0.1290 | 0.17 (0.01~2.29) | 0.1833 |
|  | Quartile4 | 12 | 119 | 0.45 (0.22~0.90) | | 0.0246 | 0.51 (0.25~1.03) | 0.0604 |
|  | Quartile3 | 15 | 97 | 0.68 (0.35~1.33) | | 0.2612 | 1.49 (0.65~3.38) | 0.3450 |
|  | Quartile2 | 17 | 121 | 0.62 (0.33~1.17) | | 0.1408 | 5.73 (2.87~11.48) | 8.05×10^-7^ |
|  | Quartile1 | 33 | 146 |  | |  |  |  |
| *Dialister* | Continuous | 77 | 483 | 3.38 (1.24~9.16) | | 0.0169 | 3.79 (1.27~11.29) | 0.0170 |
|  | Quartile4 | 24 | 121 | 1.81 (0.91~3.61) | | 0.0914 | 0.49 (0.24~1.00) | 0.0494 |
|  | Quartile3 | 22 | 117 | 1.72 (0.85~3.46) | | 0.1306 | 1.43 (0.62~3.30) | 0.3976 |
|  | Quartile2 | 16 | 108 | 1.35 (0.64~2.86) | | 0.4283 | 6.01 (3.00~12.03) | 4.16×10^-7^ |
|  | Quartile1 | 15 | 137 |  | |  |  |  |
| *Eubacterium_g10* | Continuous | 77 | 483 | 1.24 (0.54~2.84) | | 0.6186 | 1.18 (0.50~2.78) | 0.7023 |
|  | Quartile4 | 18 | 113 | 0.80 (0.42~1.54) | | 0.5082 | 0.49 (0.24~1.00) | 0.0502 |
|  | Quartile3 | 21 | 121 | 0.87 (0.47~1.64) | | 0.6744 | 1.55 (0.68~3.52) | 0.2932 |
|  | Quartile2 | 12 | 118 | 0.51 (0.25~1.06) | | 0.0718 | 5.59 (2.81~11.12) | 9.09×10^-7^ |
|  | Quartile1 | 26 | 131 |  | |  |  |  |
| *Fusobacterium* | Continuous | 77 | 483 | 0.88 (0.80~0.98) | | 0.0210 | 0.88 (0.80~0.98) | 0.0235 |
|  | Quartile4 | 12 | 121 | 0.41 (0.20~0.85) | | 0.0161 | 0.53 (0.26~1.08) | 0.0819 |
|  | Quartile3 | 15 | 119 | 0.53 (0.27~1.03) | | 0.0612 | 1.49 (0.65~3.41) | 0.3438 |
|  | Quartile2 | 21 | 122 | 0.72 (0.39~1.33) | | 0.2917 | 5.70 (2.85~11.39) | 8.34×10^-7^ |
|  | Quartile1 | 29 | 121 |  | |  |  |  |
| *Lactobacillus* | Continuous | 77 | 483 | 18.64 (1.36~254.47) | | 0.0283 | 14.85 (0.96~228.73) | 0.0532 |
|  | ≥0 | 36 | 130 | 2.38 (1.46~3.89) | | 0.0005 | 5.82 (2.91~11.61) | 6.04×10^-7^ |
|  | <0 | 41 | 353 |  | |  |  |  |
| *Lautropia* | Continuous | 77 | 483 | 0.59 (0.41~0.84) | | 0.0034 | 0.59 (0.42~0.85) | 0.0045 |
|  | Quartile4 | 5 | 121 | 0.13 (0.05~0.33) | | 2.50×10^-5^ | 0.46 (0.22~0.95) | 0.0372 |
|  | Quartile3 | 13 | 119 | 0.33 (0.17~0.65) | | 0.0014 | 1.29 (0.55~3.01) | 0.5556 |
|  | Quartile2 | 19 | 121 | 0.48 (0.26~0.87) | | 0.0164 | 5.93 (2.94~11.99) | 7.03×10^-7^ |
|  | Quartile1 | 40 | 122 |  | |  |  |  |
| *Leptotrichia* | Continuous | 77 | 483 | 0.72 (0.53~0.97) | | 0.0287 | 0.69 (0.51~0.93) | 0.0164 |
|  | Quartile4 | 12 | 121 | 0.55 (0.26~1.17) | | 0.1217 | 0.50 (0.25~1.02) | 0.0569 |
|  | Quartile3 | 24 | 116 | 1.16 (0.62~2.18) | | 0.6514 | 1.58 (0.70~3.60) | 0.2731 |
|  | Quartile2 | 19 | 123 | 0.86 (0.45~1.68) | | 0.6646 | 5.83 (2.91~11.68) | 6.38×10^-7^ |
|  | Quartile1 | 22 | 123 |  | |  |  |  |
| *Megasphaera* | Continuous | 77 | 483 | 2.50 (1.79~3.48) | | 6.26×10^-8^ | 2.24 (1.56~3.22) | 1.18×10^-5^ |
|  | Quartile4 | 44 | 120 | 6.14 (2.78~13.57) | | 7.17×10^-6^ | 0.55 (0.26~1.16) | 0.1193 |
|  | Quartile3 | 17 | 120 | 2.37 (0.99~5.70) | | 0.0531 | 1.21 (0.51~2.86) | 0.6681 |
|  | Quartile2 | 8 | 109 | 1.23 (0.45~3.38) | | 0.6892 | 5.36 (2.61~10.99) | 4.57×10^-6^ |
|  | Quartile1 | 8 | 134 |  | |  |  |  |
| *Mitsuokella* | Continuous | 77 | 483 | 12148.99 (1.38~1.07×10^8^) | | 0.0424 | 2665.38 (0.59~1.9×10^7^) | 0.0659 |
|  | ≥0 | 25 | 93 | 2.02 (1.19~3.42) | | 0.0092 | 5.80 (2.91~11.55) | 5.66×10^-7^ |
|  | <0 | 52 | 390 |  | |  |  |  |
| *Mycoplasma_g17* | Continuous | 77 | 483 | 5.76 (0.84~39.65) | | 0.0754 | 3.89 (0.47~32.13) | 0.2077 |
|  | ≥0 | 19 | 53 | 2.66 (1.47~4.80) | | 0.0012 | 5.66 (2.84~11.26) | 7.91×10^-7^ |
|  | <0 | 58 | 430 |  | |  |  |  |
| *Neisseria* | Continuous | 77 | 483 | 0.97 (0.95~1.00) | | 0.0221 | 0.98 (0.95~1.00) | 0.0484 |
|  | Quartile4 | 13 | 121 | 0.45 (0.22~0.90) | | 0.0249 | 0.48 (0.24~0.98) | 0.0435 |
|  | Quartile3 | 15 | 120 | 0.52 (0.27~1.02) | | 0.0578 | 1.27 (0.55~2.95) | 0.5743 |
|  | Quartile2 | 20 | 121 | 0.69 (0.37~1.29) | | 0.2424 | 6.05 (3.03~12.08) | 3.33×10^-7^ |
|  | Quartile1 | 29 | 121 |  | |  |  |  |
| *Oribacterium* | Continuous | 77 | 483 | 0.23 (0.08~0.66) | | 0.0059 | 0.21 (0.07~0.62) | 0.0049 |
|  | Quartile4 | 6 | 117 | 0.27 (0.11~0.68) | | 0.0056 | 0.49 (0.24~1.02) | 0.0561 |
|  | Quartile3 | 23 | 123 | 0.99 (0.53~1.83) | | 0.9677 | 1.62 (0.70~3.71) | 0.2576 |
|  | Quartile2 | 23 | 111 | 1.09 (0.59~2.03) | | 0.7763 | 5.70 (2.83~11.46) | 1.09×10^-6^ |
|  | Quartile1 | 25 | 132 |  | |  |  |  |
| *PAC000661_g* | Continuous | 77 | 483 | 5.54 (1.65~18.57) | | 0.0055 | 5.69 (1.59~20.34) | 0.0075 |
|  | Quartile4 | 28 | 120 | 2.51 (1.22~5.16) | | 0.0124 | 0.47 (0.23~0.95) | 0.0369 |
|  | Quartile3 | 14 | 102 | 1.48 (0.65~3.33) | | 0.3487 | 1.39 (0.61~3.21) | 0.4334 |
|  | Quartile2 | 23 | 132 | 1.87 (0.89~3.92) | | 0.0960 | 6.30 (3.11~12.75) | 3.09×10^-7^ |
|  | Quartile1 | 12 | 129 |  | |  |  |  |
| *Pasteurellaceae_uc* | Continuous | 77 | 483 | 0.96 (0.74~1.23) | | 0.7233 | 0.97 (0.75~1.25) | 0.7977 |
|  | Quartile4 | 17 | 121 | 0.81 (0.41~1.61) | | 0.5468 | 0.48 (0.23~0.97) | 0.0422 |
|  | Quartile3 | 13 | 117 | 0.64 (0.31~1.34) | | 0.2356 | 1.53 (0.67~3.51) | 0.3173 |
|  | Quartile2 | 26 | 124 | 1.21 (0.65~2.26) | | 0.5546 | 5.89 (2.95~11.75) | 5.07×10^-7^ |
|  | Quartile1 | 21 | 121 |  | |  |  |  |
| *Peptostreptococcus* | Continuous | 77 | 483 | 0.01 (0.00~0.64) | | 0.0320 | 0.03 (0.00~2.94) | 0.1374 |
|  | Quartile4 | 9 | 117 | 0.40 (0.18~0.87) | | 0.0217 | 0.49 (0.24~1.01) | 0.0537 |
|  | Quartile3 | 16 | 113 | 0.74 (0.38~1.41) | | 0.3584 | 1.39 (0.61~3.17) | 0.4398 |
|  | Quartile2 | 22 | 97 | 1.18 (0.64~2.16) | | 0.5934 | 5.69 (2.85~11.35) | 8.18×10^-7^ |
|  | Quartile1 | 30 | 156 |  | |  |  |  |
| *Prevotella* | Continuous | 77 | 483 | 1.02 (1.00~1.04) | | 0.0666 | 1.02 (1.00~1.04) | 0.0972 |
|  | Quartile4 | 23 | 121 | 1.35 (0.69~2.66) | | 0.3805 | 0.51 (0.25~1.03) | 0.0591 |
|  | Quartile3 | 21 | 120 | 1.25 (0.63~2.48) | | 0.5313 | 1.52 (0.67~3.45) | 0.3196 |
|  | Quartile2 | 16 | 121 | 0.94 (0.45~1.95) | | 0.8703 | 5.85 (2.94~11.66) | 5.05×10^-7^ |
|  | Quartile1 | 17 | 121 |  | |  |  |  |
| *Ralstonia* | Continuous | 77 | 483 | 9.75×10^7^ (6.57×10^4^ ~1.45×10^11^) | | 7.91×10^-7^ | 3.40×10^8^ (7.63×10^4^~1.51×10^12^) | 4.59×10^-6^ |
|  | ≥0 | 40 | 137 | 2.73 (1.67~4.45) | | 0.0001 | 6.00 (2.97~12.15) | 6.23×10^-7^ |
|  | <0 | 37 | 346 |  | |  |  |  |
| *Saccharimonas* | Continuous | 77 | 483 | 1.15 (0.93~1.42) | | 0.1955 | 1.11 (0.88~1.40) | 0.3619 |
|  | Quartile4 | 22 | 121 | 1.52 (0.75~3.06) | | 0.2462 | 0.49 (0.24~0.99) | 0.0479 |
|  | Quartile3 | 15 | 119 | 1.05 (0.49~2.24) | | 0.8988 | 1.63 (0.71~3.76) | 0.2489 |
|  | Quartile2 | 25 | 118 | 1.77 (0.89~3.51) | | 0.1052 | 5.71 (2.86~11.40) | 7.71×10^-7^ |
|  | Quartile1 | 15 | 125 |  | |  |  |  |
| *Selenomonas* | Continuous | 77 | 483 | 0.97 (0.64~1.48) | | 0.8976 | 0.95 (0.61~1.48) | 0.8271 |
|  | Quartile4 | 18 | 119 | 0.87 (0.44~1.72) | | 0.6913 | 0.48 (0.24~0.99) | 0.0458 |
|  | Quartile3 | 15 | 121 | 0.71 (0.35~1.45) | | 0.3522 | 1.67 (0.73~3.83) | 0.2284 |
|  | Quartile2 | 23 | 122 | 1.09 (0.57~2.07) | | 0.8008 | 5.76 (2.89~11.50) | 6.58×10^-7^ |
|  | Quartile1 | 21 | 121 |  | |  |  |  |

a, The number of subjects in each category. b, Odd ratio: quantifies the strength of the association between case and control. c. The multivariable ordinal logistic regression model initially included age, sex, BMI, smoking and drinking status. d, The p value is the significance of the regression coefficient. e, 25% from smallest to largest of numbers. F, between 25.1% and 50% till median. G, 51% to 75% (above the median). h, 25% of largest numbers.

**Table S5.** Relative abundances of genus-level microbiota were compared between *H. pylori* positive and negative group

| **Genus** | ***H.pylori***  **Positive**  **(n=74)** | ***H.pylori***  **Negative**  **(n=33)** | ***p*-value^a^** | **Up/down**  **(Positive/negative)** |
| --- | --- | --- | --- | --- |
| *Lautropia* | 0.6600±1.1543 | 0.6149±1.1284 | 0.0553 | Up |
| *Ralstonia* | 0.0454±0.0696 | 0.0274±0.0360 | 0.4994 | Up |
| *Anaerovorax* | 0.0060±0.0180 | 0.0037±0.0180 | 0.5018 | Up |
| *Neisseria* | 17.159±12.406 | 15.819±12.975 | 0.5528 | Up |
| *Pseudomonas* | 0.0016±0.0041 | 0.0018±0.0064 | 0.5803 | Down |
| *Megasphaera* | 0.8207±0.9204 | 0.7493±0.8507 | 0.6129 | Up |
| *Peptostreptococcus* | 0.0454±0.0696 | 0.0307±0.0255 | 0.6571 | Up |
| *Fusobacterium* | 3.8093±2.3592 | 3.8607±2.6705 | 0.9409 | Down |

a. Statistical differences were calculated by Wilcoxon rank sum test

**Table S6.** Relative abundances of genus-level microbiota were compared between tumor marker status all negative and ≥ 1 positive group

| **Genus** | **Marker status**  **≥1 positive (+)**  **(n=44)** | **Marker status**  **all negative (-)**  **(n=69)** | ***p*-value^a^** | **Up/down**  **(≥1 positive/**  **all negative)** |
| --- | --- | --- | --- | --- |
| *Lautropia* | 0.6198±0.9932 | 0.6492±1.1926 | 0.8946 | Down |
| *Ralstonia* | 0.0271±0.0433 | 0.0472±0.0680 | 0.0793 | Down |
| *Anaerovorax* | 0.0025±0.0102 | 0.0068±0.0209 | 0.3734 | Down |
| *Neisseria* | 15.478±12.340 | 16.665±12.656 | 0.6126 | Down |
| *Pseudomonas* | 0.0011±0.0039 | 0.0018±0.0053 | 0.4929 | Down |
| *Megasphaera* | 0.8577±0.8922 | 0.7589±0.8778 | 0.4617 | Up |
| *Peptostreptococcus* | 0.0381±0.0678 | 0.0412±0.0519 | 0.0456 | Down |
| *Fusobacterium* | 3.2508±1.8539 | 4.1096±2.6472 | 0.1201 | Up |

a. Statistical differences were calculated by Wilcoxon rank sum test

**Table S7.** Results of comparison KEGG pathway and orthology between GC and HC

| Pathway | | | | | |
| --- | --- | --- | --- | --- | --- |
| Pathway ID | Pathway Name | Fold change | log2FC | *p*-value | -log10  FDR |
| ko00943 | Isoflavonoid biosynthesis | 1.347 | 0.129 | 0.004 | 2.405 |
| ko05216 | Thyroid cancer | 1.236 | 0.092 | 0.011 | 1.948 |
| ko04340 | Hedgehog signaling pathway | 1.220 | 0.086 | 0.000 | 3.968 |
| ko05217 | Basal cell carcinoma | 1.206 | 0.081 | 0.000 | 3.342 |
| ko05414 | Dilated cardiomyopathy (DCM) | 1.196 | 0.078 | 0.002 | 2.640 |
| ko04341 | Hedgehog signaling pathway - fly | 1.173 | 0.069 | 0.002 | 2.621 |
| ko04512 | ECM-receptor interaction | 1.049 | 0.021 | 0.043 | 1.370 |
| ko00040 | Pentose and glucuronate interconversions | 1.038 | 0.016 | 0.002 | 2.814 |
| ko04973 | Carbohydrate digestion and absorption | 1.029 | 0.012 | 0.004 | 2.411 |
| ko04139 | Mitophagy - yeast | 1.019 | 0.008 | 0.009 | 2.070 |
| ko00983 | Drug metabolism - other enzymes | 1.014 | 0.006 | 0.007 | 2.157 |
| ko00561 | Glycerolipid metabolism | 1.014 | 0.006 | 0.002 | 2.626 |
| ko00473 | D-Alanine metabolism | 1.013 | 0.005 | 0.037 | 1.434 |
| ko01120 | Microbial metabolism in diverse environments | 0.996 | -0.002 | 0.004 | 2.350 |
| ko05132 | Salmonella infection | 0.984 | -0.007 | 0.014 | 1.855 |
| ko00920 | Sulfur metabolism | 0.982 | -0.008 | 0.010 | 1.996 |
| ko00630 | Glyoxylate and dicarboxylate metabolism | 0.980 | -0.009 | 0.008 | 2.097 |
| ko00860 | Porphyrin and chlorophyll metabolism | 0.974 | -0.011 | 0.002 | 2.780 |
| ko04214 | Apoptosis - fly | 0.960 | -0.018 | 0.000 | 6.717 |
| ko04750 | Inflammatory mediator regulation of TRP channels | 0.948 | -0.023 | 0.005 | 2.330 |
| ko04976 | Bile secretion | 0.937 | -0.028 | 0.038 | 1.417 |
| ko05034 | Alcoholism | 0.935 | -0.029 | 0.017 | 1.780 |
| ko00472 | D-Arginine and D-ornithine metabolism | 0.926 | -0.034 | 0.031 | 1.515 |
| ko04392 | Hippo signaling pathway - multiple species | 0.924 | -0.034 | 0.025 | 1.594 |
| ko05416 | Viral myocarditis | 0.921 | -0.036 | 0.050 | 1.303 |
| ko00533 | Glycosaminoglycan biosynthesis - keratan sulfate | 0.891 | -0.050 | 0.036 | 1.442 |
| ko04215 | Apoptosis - multiple species | 0.799 | -0.098 | 0.006 | 2.234 |
| ko04060 | Cytokine-cytokine receptor interaction | 0.773 | -0.112 | 0.001 | 3.042 |
| ko04950 | Maturity onset diabetes of the young | 0.697 | -0.157 | 0.000 | 3.835 |
| Orthology | | | | | |
| Orthology ID | Otrhology Name | Fold change | log2FC | *p*-value | -log10  FDR |
| K16949 | amine sulfotransferase | 11.001 | 1.041 | 4.E-10 | 9.445 |
| K11010 | superantigen YpmA | 9.444 | 0.975 | 6.E-14 | 13.235 |
| K09239 | human immunodeficiency virus type I enhancer-binding protein | 9.163 | 0.962 | 2.E-12 | 11.775 |
| K07190 | phosphorylase kinase alpha/beta subunit | 5.512 | 0.741 | 2.E-11 | 10.677 |
| K07331 | archaeal flagellar protein FlaH | 5.316 | 0.726 | 4.E-11 | 10.401 |
| K09385 | forkhead box protein G | 5.280 | 0.723 | 2.E-04 | 3.736 |
| K05304 | sialic acid synthase | 5.226 | 0.718 | 6.E-12 | 11.238 |
| K13309 | dTDP-4-amino-4,6-dideoxy-D-glucose ammonia-lyase | 5.163 | 0.713 | 6.E-06 | 5.190 |
| K04112 | benzoyl-CoA reductase subunit C | 5.135 | 0.711 | 1.E-09 | 8.976 |
| K08850 | aurora kinase, other | 4.926 | 0.693 | 2.E-06 | 5.795 |
| K15328 | bacillaene synthase trans-acting acyltransferase | 4.918 | 0.692 | 1.E-09 | 8.947 |
| K13265 | vestitone reductase | 4.485 | 0.652 | 3.E-14 | 13.520 |
| K12767 | serine/threonine-protein kinase RIM15 | 4.299 | 0.633 | 1.E-09 | 9.018 |
| K18927 | LysR family transcriptional regulator, L-lactate utilization regulator | 4.278 | 0.631 | 2.E-09 | 8.643 |
| K15479 | guanine nucleotide exchange protein RalF | 4.268 | 0.630 | 1.E-07 | 6.913 |
| K08291 | G protein-coupled receptor kinase | 4.264 | 0.630 | 8.E-13 | 12.114 |
| K11052 | CylE protein | 4.209 | 0.624 | 7.E-04 | 3.175 |
| K11053 | CylF protein | 4.209 | 0.624 | 7.E-04 | 3.175 |
| K11054 | CylI protein | 4.209 | 0.624 | 7.E-04 | 3.175 |
| K06499 | carcinoembryonic antigen-related cell adhesion molecule | 4.129 | 0.616 | 8.E-05 | 4.093 |
| K19813 | glucose dehydrogenase | 0.399 | -0.399 | 4.E-06 | 5.391 |
| K17226 | sulfur-oxidizing protein SoxY | 0.396 | -0.402 | 5.E-06 | 5.345 |
| K15651 | aminocarboxycyclopropane-forming enzyme | 0.394 | -0.404 | 8.E-07 | 6.075 |
| K08983 | putative membrane protein | 0.394 | -0.405 | 9.E-07 | 6.061 |
| K15645 | coronafacic acid polyketide synthase Cfa7 | 0.393 | -0.406 | 1.E-07 | 7.008 |
| K07180 | serine protein kinase | 0.391 | -0.408 | 9.E-06 | 5.053 |
| K05563 | multicomponent K+:H+ antiporter subunit F | 0.386 | -0.413 | 6.E-08 | 7.244 |
| K07169 | FHA domain-containing protein | 0.382 | -0.417 | 3.E-07 | 6.497 |
| K08948 | chlorosome envelope protein D | 0.379 | -0.422 | 3.E-02 | 1.461 |
| K17292 | tubulin-specific chaperone A | 0.373 | -0.428 | 2.E-07 | 6.609 |
| K05564 | multicomponent K+:H+ antiporter subunit G | 0.370 | -0.432 | 7.E-08 | 7.175 |
| K05561 | multicomponent K+:H+ antiporter subunit D | 0.370 | -0.432 | 6.E-08 | 7.213 |
| K09711 | uncharacterized protein | 0.369 | -0.433 | 2.E-04 | 3.804 |
| K16517 | porin-like protein GalP | 0.363 | -0.440 | 3.E-07 | 6.533 |
| K05504 | growth differentiation factor 15 | 0.359 | -0.445 | 2.E-07 | 6.603 |
| K12408 | cholest-5-ene-3beta,7alpha-diol 3beta-dehydrogenase | 0.359 | -0.445 | 6.E-08 | 7.235 |
| K05502 | bone morphogenetic protein 1 | 0.359 | -0.445 | 3.E-07 | 6.571 |
| K05508 | C-X3-C motif chemokine 1 | 0.358 | -0.446 | 2.E-07 | 6.647 |
| K05497 | growth differentiation factor 8 | 0.358 | -0.447 | 3.E-07 | 6.523 |
| K13585 | holdfast attachment protein HfaA | 0.354 | -0.451 | 3.E-05 | 4.518 |
| K07782 | LuxR family transcriptional regulator, quorum-sensing system regulator SdiA | 0.354 | -0.451 | 3.E-07 | 6.593 |
| K10290 | F-box protein 3 | 0.353 | -0.452 | 2.E-03 | 2.725 |
| K08399 | leucine-rich repeat-containing G protein-coupled receptor 6 | 0.352 | -0.454 | 1.E-04 | 3.881 |
| K15305 | vacuole morphology and inheritance protein 14 | 0.351 | -0.455 | 1.E-07 | 6.840 |
| K18580 | rhamnogalacturonan hydrolase | 0.343 | -0.464 | 4.E-03 | 2.430 |
| K09866 | aquaporin-4 | 0.343 | -0.465 | 7.E-08 | 7.143 |
| K11439 | type I protein arginine methyltransferase | 0.340 | -0.469 | 1.E-03 | 2.868 |
| K10236 | trehalose/maltose transport system substrate-binding protein | 0.339 | -0.470 | 5.E-08 | 7.281 |
| K17322 | glycerol transport system permease protein | 0.334 | -0.476 | 2.E-07 | 6.694 |
| K17756 | long-chain-alcohol oxidase | 0.331 | -0.480 | 3.E-08 | 7.567 |
| K07401 | selenoprotein W-related protein | 0.331 | -0.480 | 2.E-08 | 7.704 |
| K09362 | homeo domain only family, other | 0.324 | -0.489 | 2.E-03 | 2.771 |
| K18552 | MFS transporter, DHA1 family, florfenicol/chloramphenicol resistance protein | 0.322 | -0.492 | 3.E-05 | 4.480 |
| K08002 | matrix metalloproteinase-24 (membrane-inserted) | 0.321 | -0.494 | 1.E-03 | 2.863 |
| K09950 | uncharacterized protein | 0.320 | -0.496 | 2.E-09 | 8.807 |
| K15141 | mediator of RNA polymerase II transcription subunit 28 | 0.295 | -0.530 | 5.E-04 | 3.345 |
| K06091 | MAGUK p55 subfamily member 5 | 0.290 | -0.537 | 6.E-04 | 3.204 |
| K09593 | GRB2-associated-binding protein 1 | 0.268 | -0.572 | 5.E-05 | 4.327 |
| K13746 | carboxynorspermidine synthase | 0.259 | -0.587 | 3.E-04 | 3.508 |
| K10253 | DOPA 4,5-dioxygenase | 0.257 | -0.590 | 9.E-08 | 7.043 |
| K11214 | sedoheptulokinase | 0.249 | -0.603 | 2.E-04 | 3.635 |
| K09328 | homeobox protein Unc-4 | 0.189 | -0.724 | 2.E-03 | 2.640 |
| K17212 | bisdemethoxycurcumin synthase | 0.178 | -0.751 | 8.E-04 | 3.094 |

FC; Fold change, FDR; False Discovery Rate

**Table S8.** List of KEGG pathway and orthology IDs with corresponding pathway names shown in the correlation analysis

| Pathway ID | Pathway Name |
| --- | --- |
| Ko05034 | Alcoholism |
| Ko00920 | Sulfur metabolism |
| Ko05416 | Viral myocarditis |
| Ko00533 | Glycosaminoglycan biosynthesis - keratan sulfate |
| Ko04976 | Bile secretion |
| Ko04750 | Inflammatory mediator regulation of TRP channels |
| Ko01120 | Microbial metabolism in diverse environments |
| Ko04950 | Maturity onset diabetes of the young |
| Ko04060 | Cytokine-cytokine receptor interaction |
| Ko04215 | Apoptosis - multiple species |
| Ko04392 | Hippo signaling pathway - multiple species |
| Ko04214 | Apoptosis - fly |
| Ko00363 | Bisphenol degradation |
| Ko05132 | Salmonella infection |
| Ko00860 | Porphyrin and chlorophyll metabolism |
| Ko00630 | Glyoxylate and dicarboxylate metabolism |
| Ko04341 | Hedgehog signaling pathway - fly |
| Ko04340 | Hedgehog signaling pathway |
| Ko05217 | Basal cell carcinoma |
| Ko05216 | Thyroid cancer |
| Ko04139 | Mitophagy - yeast |
| Ko04512 | ECM-receptor interaction |
| Ko00943 | Isoflavonoid biosynthesis |
| Ko04973 | Carbohydrate digestion and absorption |
| Ko05414 | Dilated cardiomyopathy (DCM) |
| Ko00561 | Glycerolipid metabolism |
| Ko00472 | D-Arginine and D-ornithine metabolism |
| Ko00040 | Pentose and glucuronate interconversions |
| Ko00983 | Drug metabolism - other enzymes |
| Ko00473 | D-Alanine metabolism |
| Ko05034 | Alcoholism |
| Orthology ID | Orthology Name |
| K09239 | human immunodeficiency virus type I enhancer-binding protein |
| K04112 | benzoyl-CoA reductase subunit C |
| K07331 | archaeal flagellar protein FlaH |
| K07190 | phosphorylase kinase alpha/beta subunit |
| K05508 | C-X3-C motif chemokine 1 |
| K05497 | growth differentiation factor 8 |
| K07169 | FHA domain-containing protein |
| K05504 | growth differentiation factor 15 |
| K05502 | bone morphogenetic protein 1 |
| K07782 | LuxR family transcriptional regulator, quorum-sensing system regulator SdiA |
| K09866 | aquaporin-4 |
| K09950 | uncharacterized protein |
| K07401 | selenoprotein W-related protein |
| K07180 | serine protein kinase |
| K05564 | multicomponent K+:H+ antiporter subunit G |
| K05563 | multicomponent K+:H+ antiporter subunit F |
| K05561 | multicomponent K+:H+ antiporter subunit D |
| K08983 | putative membrane protein |
| K06091 | MAGUK p55 subfamily member 5 |
| K05304 | sialic acid synthase |
| K08948 | chlorosome envelope protein D |
| K08291 | G protein-coupled receptor kinase |
| K09385 | forkhead box protein G |
| K06499 | carcinoembryonic antigen-related cell adhesion molecule |
| K08850 | aurora kinase, other |
| K09593 | GRB2-associated-binding protein 1 |
| K08399 | leucine-rich repeat-containing G protein-coupled receptor 6 |
| K09328 | homeobox protein Unc-4 |
| K08002 | matrix metalloproteinase-24 (membrane-inserted) |
| K09362 | homeo domain only family, other |
| K09711 | uncharacterized protein |


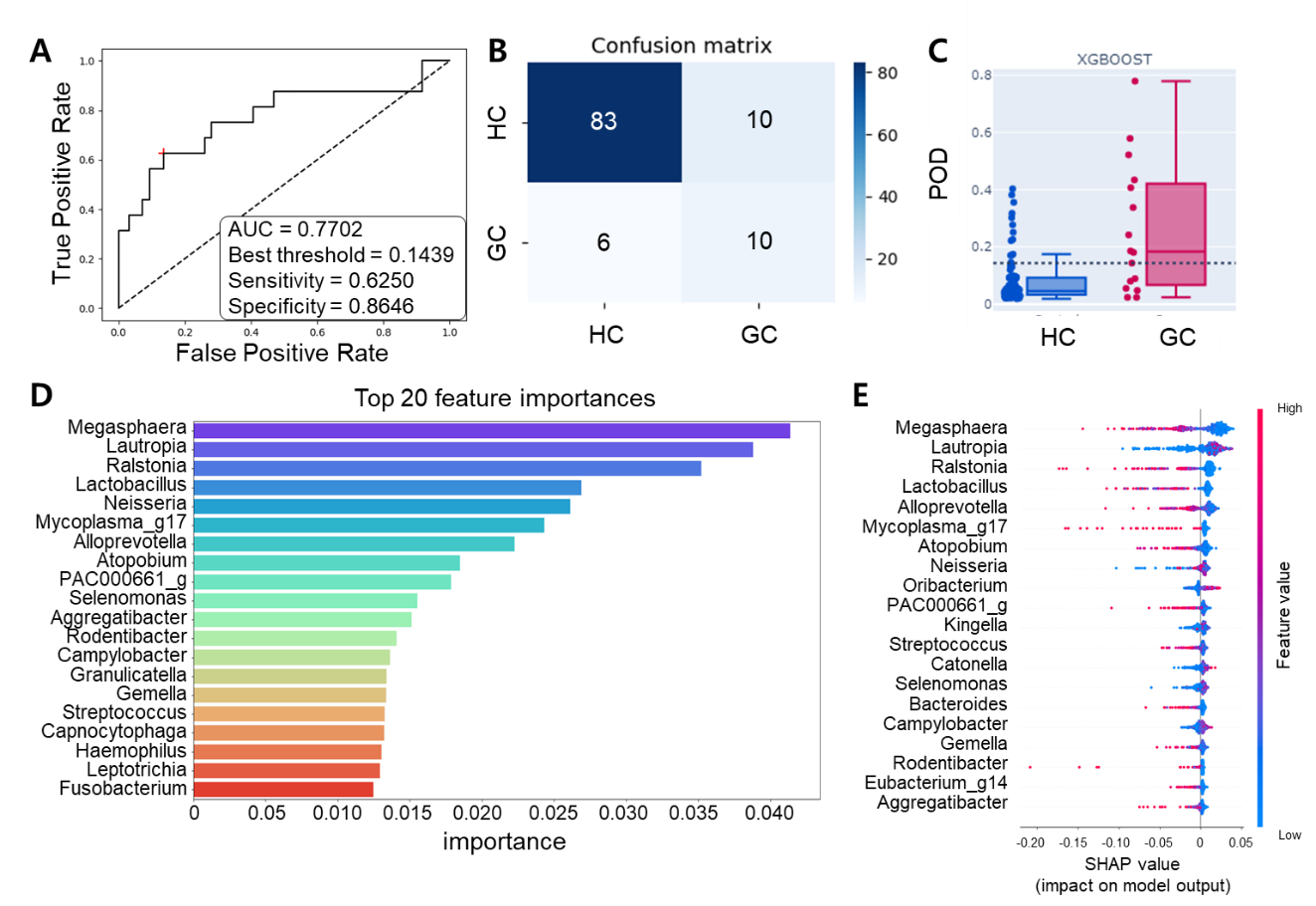


**Figure S1**. Fold 1 of XGB model. A. ROC curve. B. Confusion matrix represent model performance evaluation indicator. C. The POD graph shows the sensitivity of the model for different threshold values. D. The variable importance histogram of fold 1. E. It is a value obtained by composing a combination of various features to obtain the importance of one feature and averaging the change depending on the presence or absence of the feature.

**
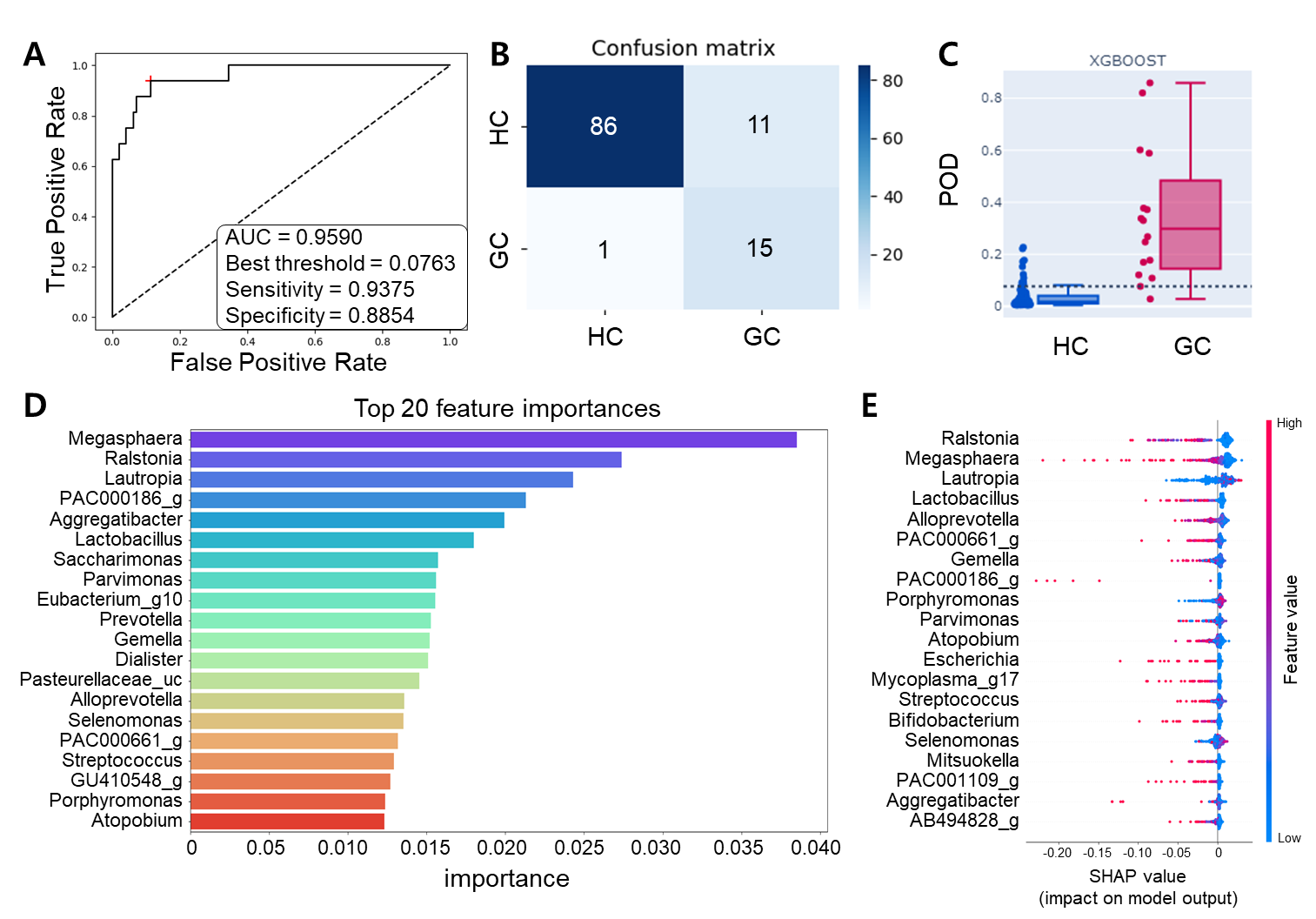
**

**Figure S2**. Fold 2 of XGB model. A. ROC curve. B. Confusion matrix represent model performance evaluation indicator. C. The POD graph shows the sensitivity of the model for different threshold values. D. The variable importance histogram of fold 2. E. It is a value obtained by composing a combination of various features to obtain the importance of one feature and averaging the change depending on the presence or absence of the feature.

**
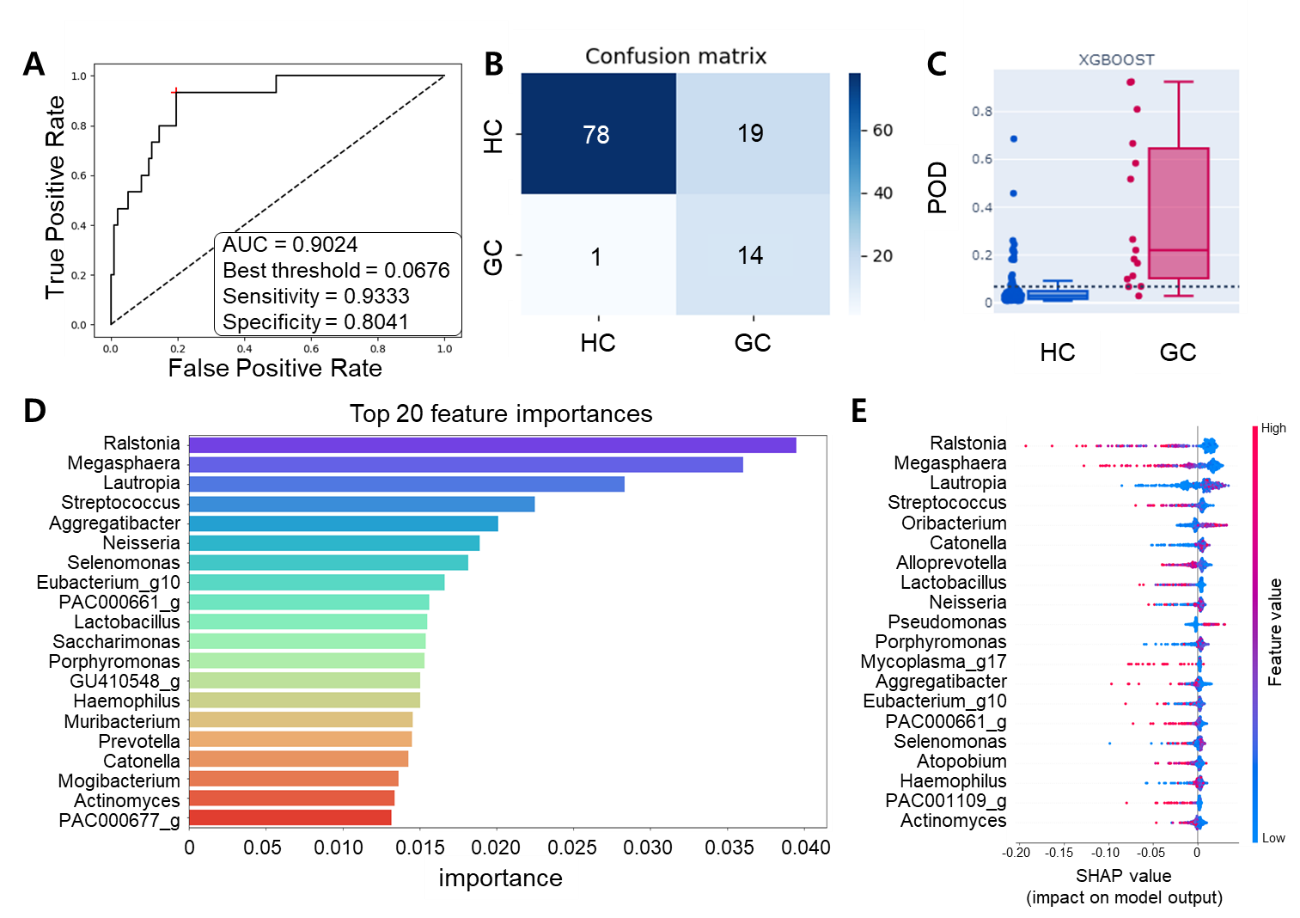
**

**Figure S3**. Fold 3 of XGB model. A. ROC curve. B. Confusion matrix represent model performance evaluation indicator. C. The POD graph shows the sensitivity of the model for different threshold values. D. The variable importance histogram of fold 3. E. It is a value obtained by composing a combination of various features to obtain the importance of one feature and averaging the change depending on the presence or absence of the feature.

**
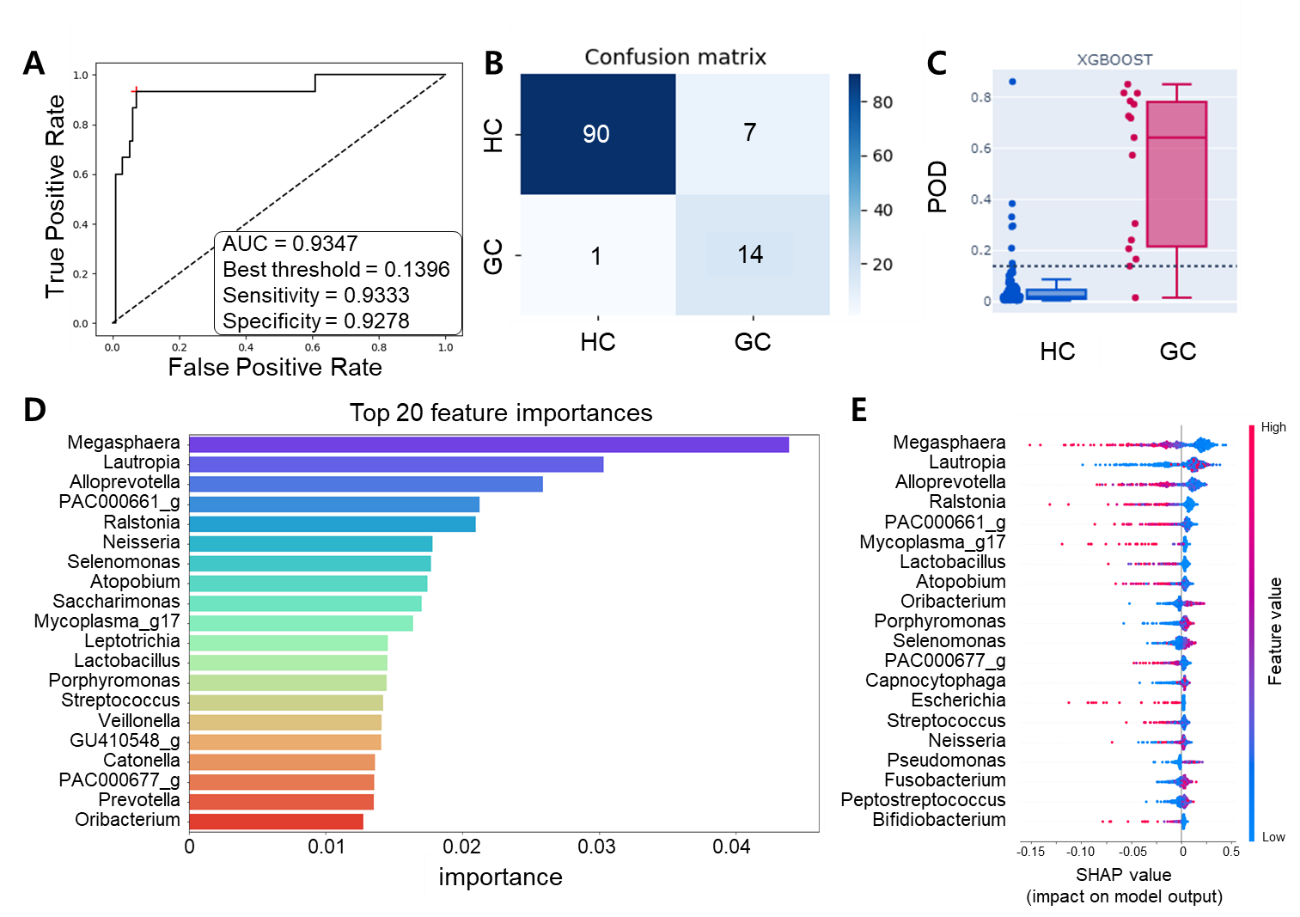
**

**Figure S4**. Fold 4 of XGB model. A. ROC curve. B. Confusion matrix represent model performance evaluation indicator. C. The POD graph shows the sensitivity of the model for different threshold values. D. The variable importance histogram of fold 4. E. It is a value obtained by composing a combination of various features to obtain the importance of one feature and averaging the change depending on the presence or absence of the feature.

**
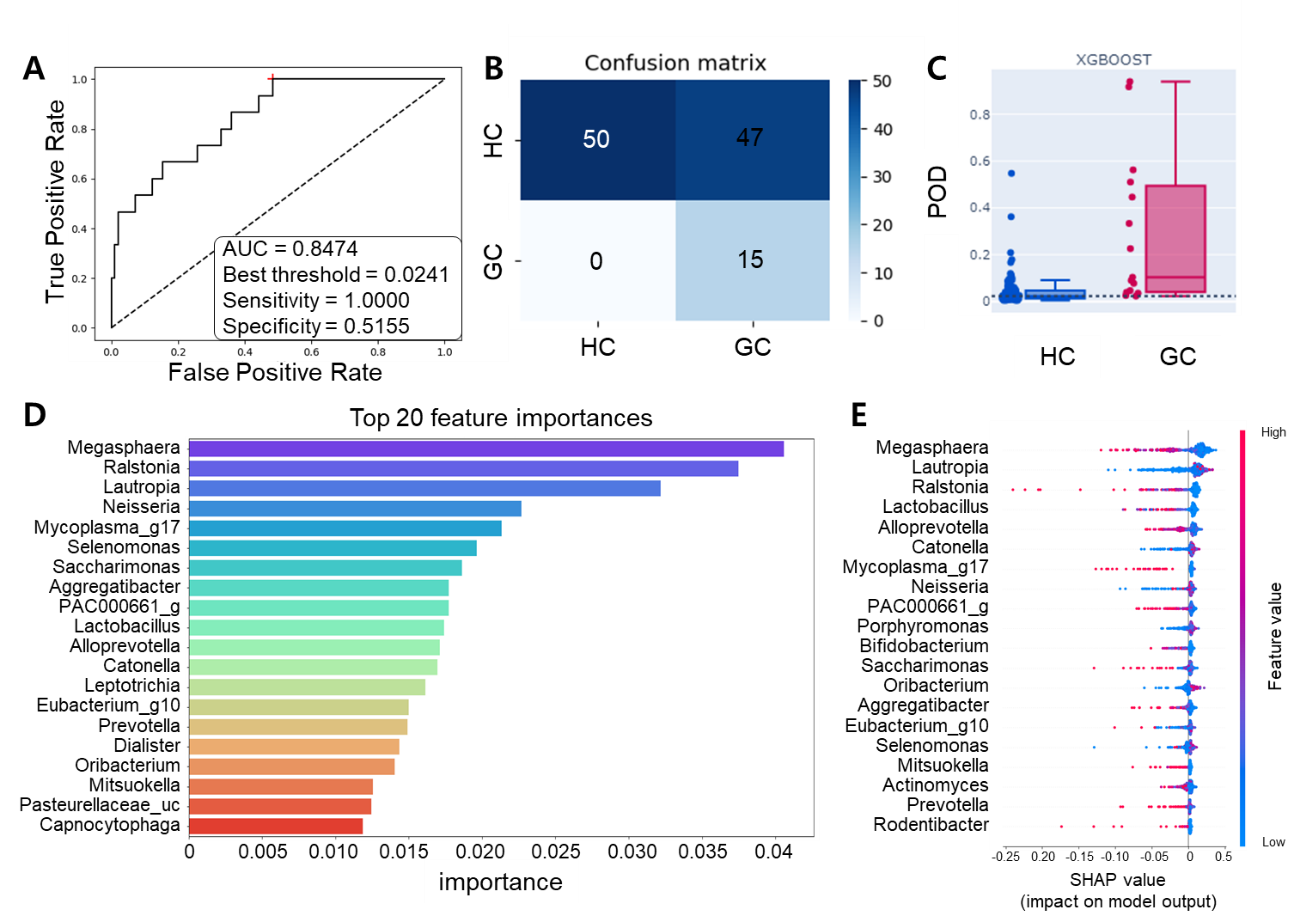
**

**Figure S5**. Fold 5 of XGB model. A. ROC curve. B. Confusion matrix represent model performance evaluation indicator. C. The POD graph shows the sensitivity of the model for different threshold values. D. The variable importance histogram of fold 5. E. It is a value obtained by composing a combination of various features to obtain the importance of one feature and averaging the change depending on the presence or absence of the feature.


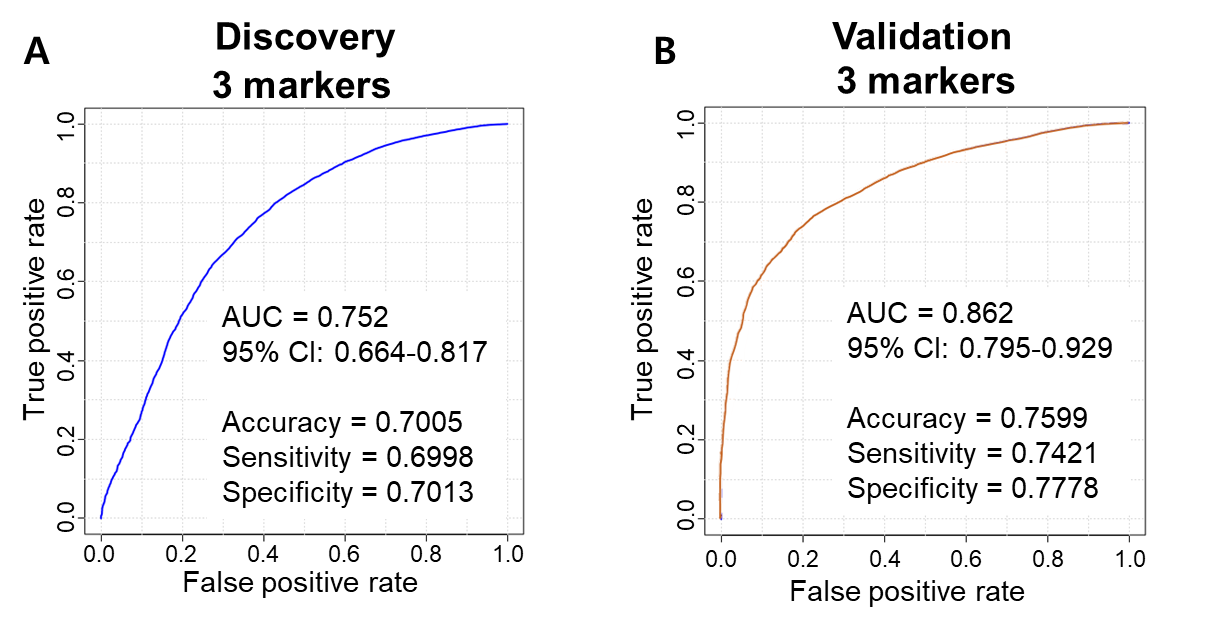


**Figure S6.** ROC curves for diagnostic performance of oral microbial markers in gastric cancer.

(A) ROC curve based on three genera (Ralstonia, Megasphaera, and Lautropia) in the discovery set. (B) ROC curve based on the same genera in the validation set.


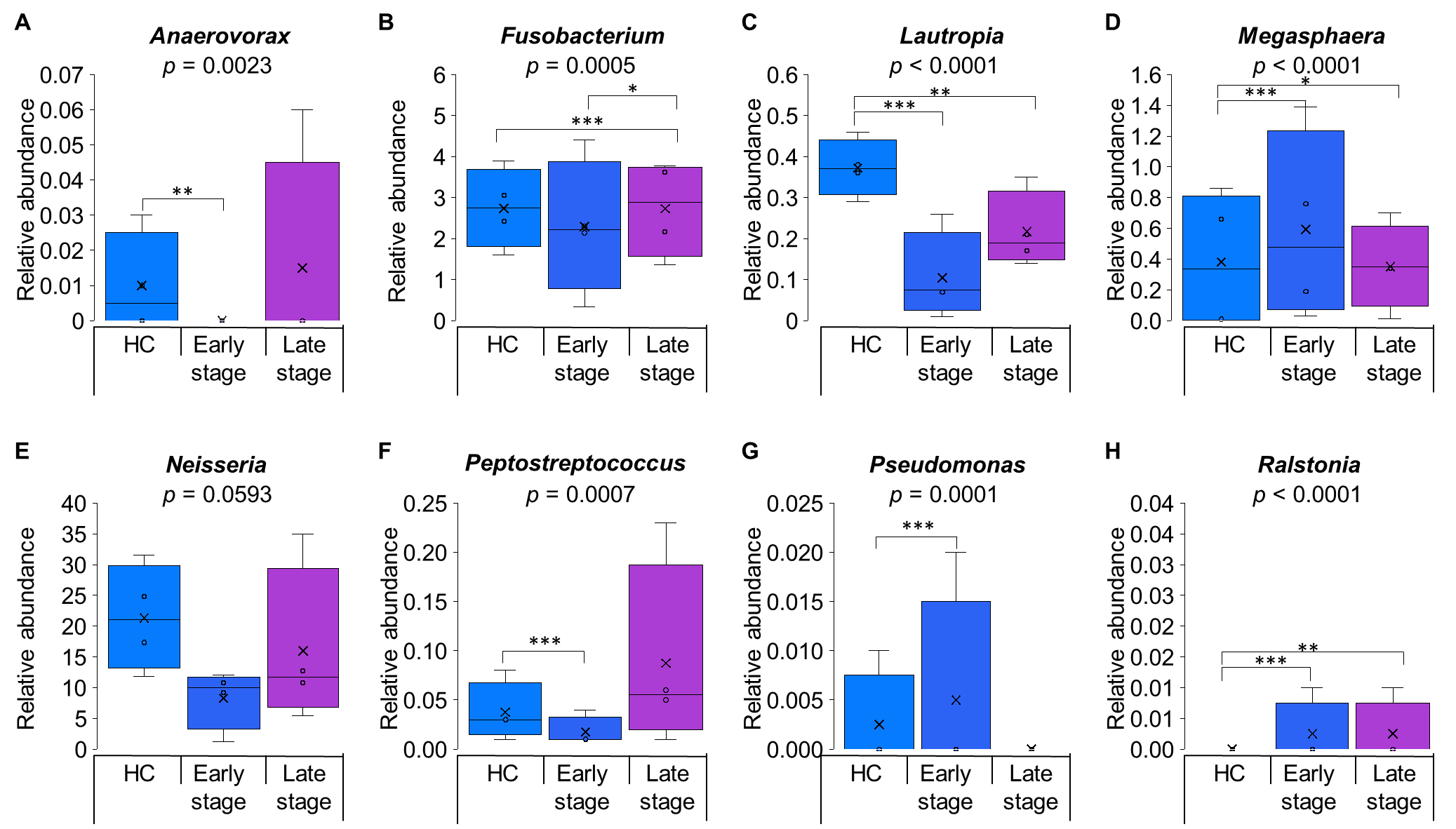


**Figure S7.** Relative abundance of eight genera in HC, early-, and late stage in gastric cancer (A) *Anaerovorax*, (B) *Fusobacterium*, (C) *Lautropia*, (D) *Megasphaera*, (E) *Neisseria*, (F) *Peptostreptococcus*, (G) *Pseudomonas*, and (H) *Ralstonia*. Statistical significance * *p* < 0.05, ** *p* < 0.01, *** *p* < 0.001


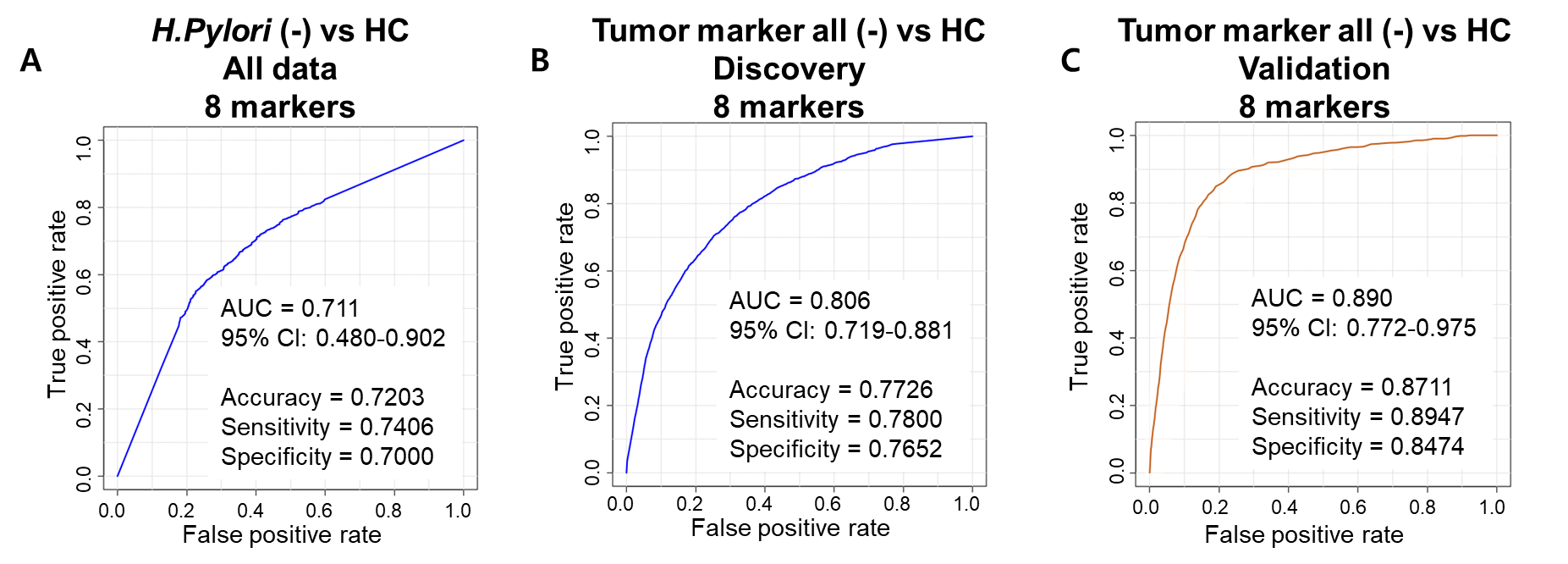


**Figure S8.** Diagnostic performance of oral microbial markers in clinical subgroups of gastric cancer.

(A) ROC curve showing the diagnostic performance of the eight selected oral microbial markers in distinguishing Helicobacter pylori-negative GC patients from HC. (B) ROC curve comparing tumor marker-negative GC patients (CEA, CA19-9, and CA72-4 all within reference range) to HC in the discovery and (C) validation set.


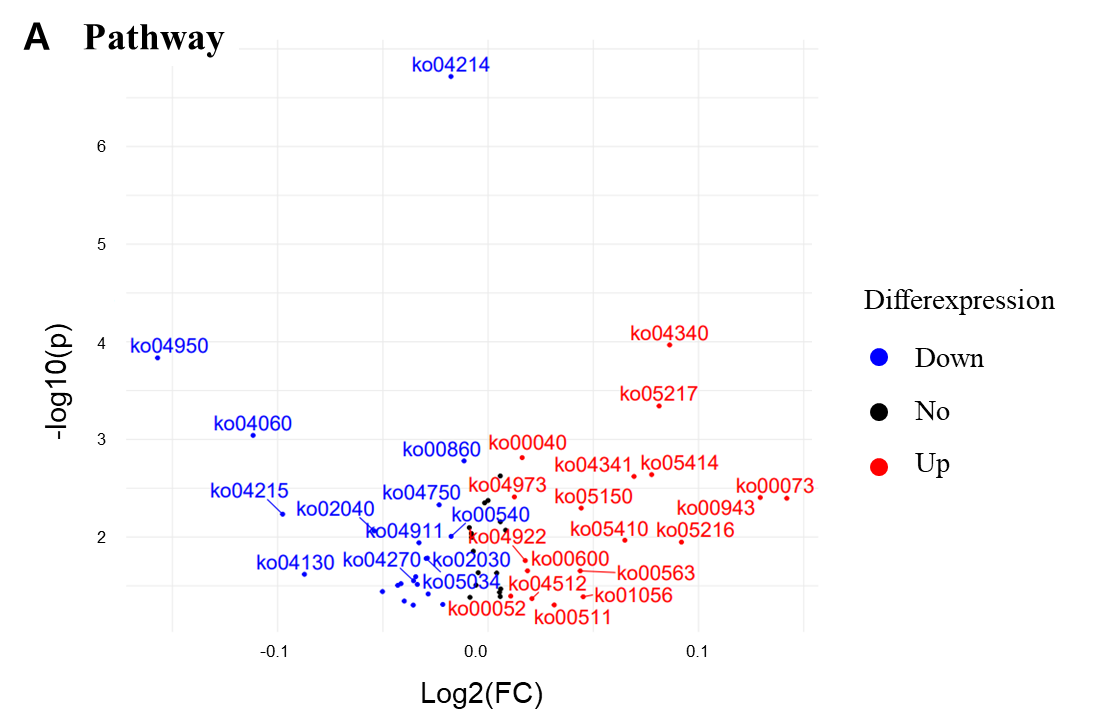


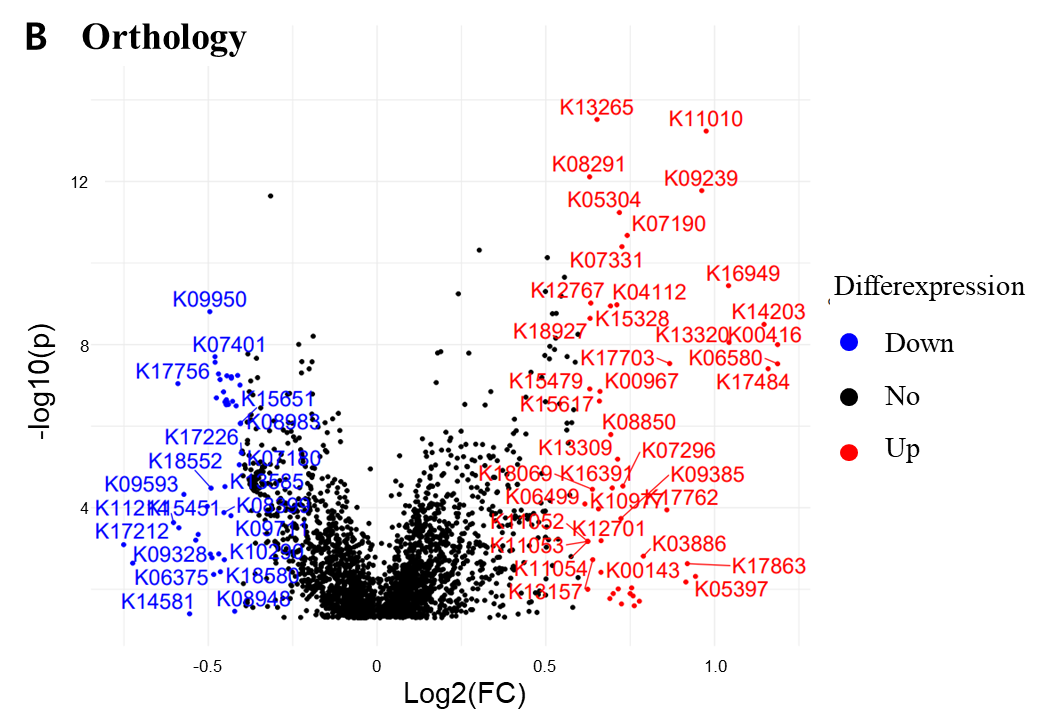


**Figure S9.** Volcano plot showing differences in KEGG pathway and orthology in GC compared with HC. The x axis denotes log2 fold change value. The y axis denotes adjusted p value that determines in Wilcoxon rank sum test. A. The up- and down-regulated KEGG pathway were determined by p value <= 0.05 and FC > 1 and < 1. B. The up- and down-regulated KEGG orthology were determined by p value <= 0.05 and FC > 4 and < 0.4.

**
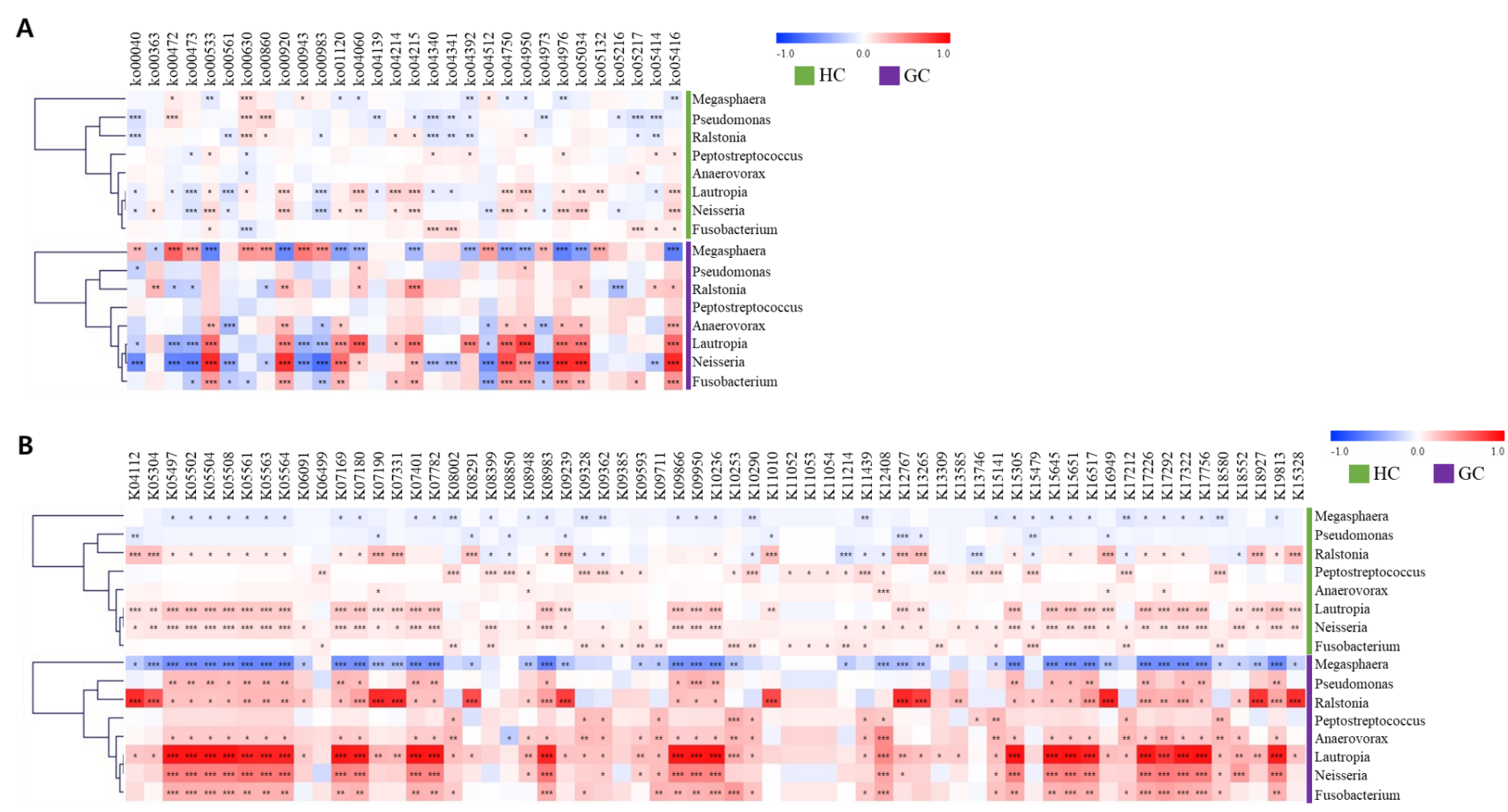
**

**Figure S10.** Correlation between genus and functional profiles. A. Heatmap of Spearman’s rank correlation analysis between 8 genera (columns) and 30 significant pathways (rows) in two groups. B. Heatmap of Spearman’s rank correlation analysis between 8 genera (columns) and 63 significant orthology (rows) in two groups. The color represents the coefficient of correlation. * means the *p* value < 0.05, ** means the *p* value < 0.01, and *** means the *p* value < 0.001.
